# Anxiety, depression and post-traumatic stress disorder in refugees resettling in high-income countries: systematic review and meta-analysis

**DOI:** 10.1192/bjo.2020.54

**Published:** 2020-07-02

**Authors:** Jens-R. Henkelmann, Sanne de Best, Carla Deckers, Katarina Jensen, Mona Shahab, Bernet Elzinga, Marc Molendijk

**Affiliations:** Faculty of Social and Behavioural Sciences, Clinical Psychology Department, Leiden University, The Netherlands; Faculty of Social and Behavioural Sciences, Clinical Psychology Department, Leiden University; Faculty of Social and Behavioural Sciences, Clinical Psychology Department, Leiden University; Faculty of Social and Behavioural Sciences, Clinical Psychology Department, Leiden University; Faculty of Social and Behavioural Sciences, Clinical Psychology Department, Leiden University; and Clinical Epidemiological Department, Leiden University Medical Center; Faculty of Social and Behavioural Sciences, Clinical Psychology Department, Leiden University; Faculty of Social and Behavioural Sciences, Clinical Psychology Department, Leiden University; and Leiden Institute of Brain and Cognition, Leiden University Medical Center, The Netherlands

**Keywords:** Refugees, mental health, depression, anxiety, PTSD

## Abstract

**Background:**

The number of refugees is at its highest since the Second World War and on the rise. Many refugees suffer from anxiety, depression and post-traumatic stress disorder (PTSD), but exact and up-to-date prevalence estimates are not available.

**Aims:**

To report the pooled prevalence of anxiety and mood disorders and PTSD in general refugee populations residing in high-income countries and to detect sources of heterogeneity therein.

**Method:**

Systematic review with meta-analyses and meta-regression.

**Results:**

Systematic searches (final search date 3 August 2019) yielded 66 eligible publications that reported 150 prevalence estimates (total sample *N* = 14 882). Prevalence rates were 13 and 42% (95% CI 8–52%) for diagnosed and self-reported anxiety, 30 and 40% (95% CI 23–48%) for diagnosed and self-reported depression, and 29 and 37% (95% CI 22–45%) for diagnosed and self-reported PTSD. These estimates are substantially higher relative to those reported in non-refugee populations over the globe and to populations living in conflict or war settings, both for child/adolescent and adult refugees. Estimates were similar over different home and resettlement areas and independent of length of residence.

**Conclusions:**

Our data indicate a challenging and persisting disease burden in refugees due to anxiety, mood disorders and PTSD. Knowing this is relevant for the development of public health policies of host countries. Scalable interventions, tailored for refugees, should become more readily available.

Refugees are people forced to flee from their home country for reasons such as war, violence or fear of persecution. According to a recent estimate, the number of forcibly displaced people is around 70 million (of whom 26 million have refugee status). This estimate is the highest since the Second World War and it is on the rise. This is partly due to the ongoing Syrian civil war, which forced millions of people to flee.^1,2^

The majority of refugees are repeatedly exposed to stress and traumatic events in their home country and during their journey to safer areas.^3^ During resettlement they often face unemployment, loneliness and uncertainty about asylum procedures^4^ and the future.^5^ Limited access to food and/or medical care is common.^6^ These factors may all contribute to the relatively high prevalence of mental disorders in refugees.^7–9^

The mental health status of refugees has been the topic of a large number of studies, but it has proven to be difficult to estimate the prevalence of mental illness in this population. The systematic reviews on this topic^10–12^ show large variations in reported prevalence rates (e.g. between 5 and 80% for depression and between 3 and 88% for post-traumatic stress disorder (PTSD)). This was recently confirmed by Morina et al,^13^ who performed a systematic review on psychiatric disability in refugees and internally displaced persons. Their results show large variations in the prevalence not only of mood and anxiety disorders, but also of alcohol dependence and psychotic symptoms. In fact, the conclusion of this study was that there is ‘a substantial lack of data concerning the wider extent of psychiatric disability among people living in protracted displacement situations’.^13^

Meta-analyses have been performed on the topic as well. In adult refugees, for instance, Fazel et al^14^ report a prevalence of 4–6% for depression (based on 14 studies) and 8–10% for PTSD (based on 17 studies). Two more recent meta-analyses including mainly adult refugees^15,16^ report substantially higher prevalence (25–45% for depression, 21–35% for anxiety disorders and 31–63% for PTSD). This difference is probably due to the inclusion of both interview and self-report assessments in the latter studies, while Fazel et al^14^ included only studies in which mental health status was assessed by means of an interview.

To obtain a better perspective on prevalence rates, between-study heterogeneity should be explained as well as understood,^17^ and this can be achieved by means of subgroup and meta-regression analyses.^18,19^ Yet, to date, few efforts have been made to understand and explain heterogeneity in prevalence rates of mental illness in refugees. Additionally, it is unknown whether the earlier reported estimates of prevalence also apply to more recent refugee movements and whether they differ as a function of country of resettlement and/or country of origin and length of residence.

Well-informed and up-to-date information on prevalence rates of mental health problems in refugees is necessary not only for a more fine-tuned assessment of risks and their needs, so that subsequent public health policies can be developed, but also to gain a more general understanding of the etiology of mental disorders. The present paper reports an updated version of previous meta-analyses^14–16^ on prevalence rates of self-reported and diagnosed anxiety disorders, depressive disorders and PTSD in general adult and child/adolescent refugee populations, resettled in high-income countries. Informed by earlier work, we have a particular interest in investigating potential sources of heterogeneity in reported prevalence rates.

## Method

This systematic review has been performed and is reported according to the guidelines and checklists set forth by MOOSE^20^ and PRISMA.^21^ A review protocol was drafted and pre-registered at PROSPERO (CRD42018100539).

### Search and selection strategy

We searched Embase, PubMed, Web of Science and Google Scholar^22^ for articles reporting on prevalence rates of depressive disorders, anxiety disorders and/or PTSD in general refugee samples. The following search string was used: ((refugee* OR displace* OR stateless*) AND ((psych* AND (disor* OR ill* OR health)). Only articles that were written in English, German, French, Spanish, Turkish, Danish or Dutch were considered. Reference lists of reviews and meta-analyses were used as additional sources of eligible articles. We also conducted a grey literature search and went through the preprint services PsyArXiv, SocArXiv and MedArXiv for eligible articles. The final search date was 3 August 2019.

A first decision on eligibility was based on the title and abstract of candidate articles. A next decision was based on the article's full text. At least two members of the review team made a final decision on the eligibility of each article, based on the inclusion and exclusion criteria provided below.

### Inclusion and exclusion criteria

Articles were included if they reported: (a) the prevalence rates of anxiety disorders, depressive disorders or PTSD as assessed according to a structured or semi-structured diagnostic interview or a validated cut-off score on a questionnaire; (b) data on refugee samples residing in countries that have reached very high human development in 2019, defined and compiled by the United Nations Development Programme^23,24^ and classified here as ‘high-income countries’; and (c) original data (i.e. reviews, for example, were excluded). Note that, due to the second inclusion criterion, internally displaced populations were not investigated here.

To include homogeneous diagnostic descriptions, notably for PTSD, papers had to be published after the publication of DSM-III-R in 1987. If two articles reported on the same data-set, we included the article that contained the most information. If an article reported the presence of mental illness on the basis of both a diagnosis and a cut-off score, we included (only) the diagnostic data.^25^

Articles were excluded if: (a) the sample reported on was not drawn from the general refugee population (e.g. articles reporting data gathered in a hospital were excluded) or (b) no relevant outcome data could be extracted from the article, even after contact with (or attempts to contact) the corresponding author of the article. The final inclusion decision for each article was based on full agreement among the members of the review team.

### Assessment of methodological quality

The methodological quality of eligible studies was independently assessed by two members of the review team (C.D. and M.M.) using the quality assessment tool for observational cohort and cross-sectional studies that is recommended by the USA National Institutes of Health.^26^

### Data extraction

From the eligible papers, at least two independent researchers extracted data on sample size, percentage of females, mean age, country of origin, host country, assessment type, prevalence rates of depressive disorders, anxiety disorders and PTSD, whether language-adapted assessments were included, and the average time of stay in the host country at time of assessment. In extracting prevalence data, we ensured that PTSD was not included in reported prevalence rates for ‘any anxiety disorder’. Following the literature in this field, we considered the depressive disorders as representing a single category. If prevalence rates were reported for multiple depressive disorders in a single sample, we aimed to pool these estimates (preferably with the help of the corresponding author of the article on the sample). If we could not come to a reasonable and single estimate, the article was excluded.

### Statistical analysis

Analyses were performed in jamovi (version 0.9)^27^ and Stata (version 13) for macOS.^28^ Summary tables on characteristics of eligible papers were created.

Random-effects meta-analyses were used to pool the data on prevalence rates. Prevalence estimates were reported together with their respective two-tailed 95% confidence intervals (CIs). We stabilised the variance by means of double arcsine transformations, which is the method of choice when outcome data are prevalence rates.^29^ For interpretational purposes, we present data that is back-transformed. Heterogeneity among studies was quantified using the *I*^2^-statistic and its statistical significance was assessed using the *Χ*^2^-statistic.^30^ If heterogeneity in outcome was present, subgroup and meta-regression analyses were performed. Predictors of heterogeneity were: mean age of the sample, percentage of females in the sample, average amount of time in the host country for the sample (in months), type of assessment (diagnosis versus cut-off score), continent of origin (Africa, Asia, Europe, and a ‘mix’ or ‘other’ category), host continent (Australia, Europe or North America), whether assessments were language adjusted/included the use of an interpreter (yes versus no) and methodological quality (as a continuous score). Publication bias was assessed by means of Kendall's tau, a rank correlation test for the assessment of funnel plot asymmetry.^30^ Statistical significance was set at *P* < 0.05.

## Results

### Study selection

**Fig. 1:**
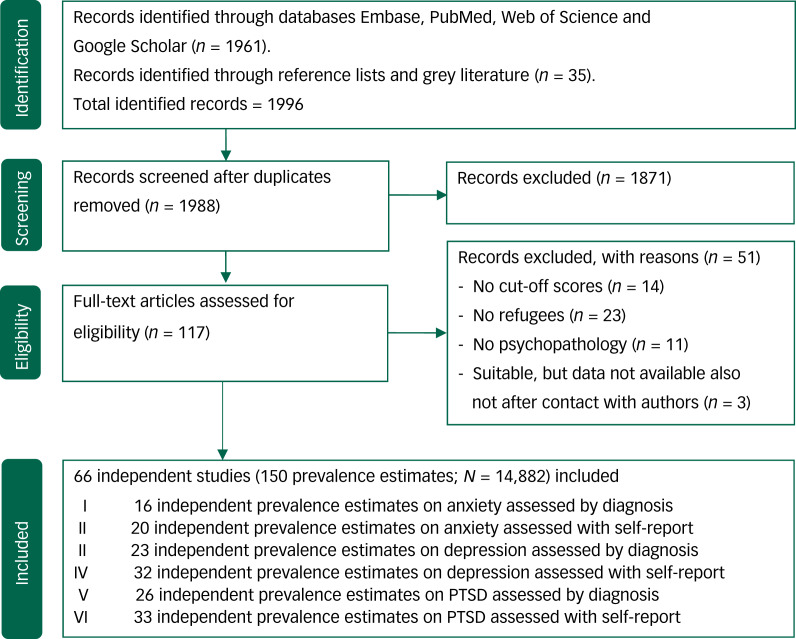
Flowchart on identification, screening and inclusion of eligible publications. PTSD, post-traumatic stress disorder.

We identified 1988 articles after removal of duplicates, of which 117 articles were deemed relevant after screening of title and abstract. After full text assessment, another 51 articles were excluded. The final number of articles that was included was 66 (total sample size *N* = 14 882, average sample size per study *n* = 225, range 6–1603). From these articles we could extract 150 prevalence estimates (*K*). Figure 1 summarises the search and selection process.

The mean age of the included samples was 33.4 years (s.d. = 12.3) and 45.8% were women. Asia (40.9%), Europe (10.6%) and Africa (9.1%) were the most frequently reported continents of origin. The most frequently reported continents of resettlement were Europe (39.4%), North America (30.3%) and Australia (24.3%). Most studies (63.6%) applied self-report measures to estimate prevalence rates of mental health problems (36.4% used diagnostic interviews). The characteristics of the studies are presented in Table 1. No studies on internally displaced populations in high-income countries were detected. Hence, as a result of the second inclusion criterion (i.e. inclusion if the refugee sample resided in a high-income country), only studies that assessed mental health in refugees (as opposed to internally displaced populations) were included. Supplementary Table 1, available at https://doi.org/10.1192/bjo.2020.54, provides additional information on the samples and applied methodology of the included articles.

**Table 1:** Characteristics of included studies and samples

Study	*n*	Mean age, years^a^	Female, %	Country of origin	Host country	Analysis^b^
Westermeyer (1988)^31^	97	37	46	Laos	USA	I, III
Hinton et al (1993)^32^	201	33	52	Vietnam	USA	I, III, V
Carlson & Rosser-Hogan (1994)^33^	50	42	52	Cambodia	USA	II, IV, VI
Cheung (1994)^34^	223	40	53	Cambodia	New Zealand	V
Pernice & Brook (1994)^35^	129	*N.K.*	*N.K.*	‘Indochina’	New Zealand	II. IV
Weine et al (1995)^36^	20	35	50	Bosnia and Herzegovina	USA	IV, VI
Malekzai et al (1996)^37^	30	42	50	Afghanistan	USA	V
D'Avanzo & Barab (1998)^38^	175	29	100	Cambodia	Mix	II, IV
Almqvist & Broberg (1999)^39^	39	8	26	Iran	Sweden	VI
Favaro et al (1999)^40^	40	31	48	Mix	Italy	III, V
Mollica et al (1999)^41^	534	50	59	Bosnia and Herzegovina	Croatia	II, VI
Sack et al (1999)^42^	30	22	33	Cambodia	USA	I, V
Tousignant et al (1999)^43^	203	16	52	Mix	Canada	I, III
Papageorgiou et al (2000)^44^	95	10	57	Bosnia and Herzegovina	Greece	II, IV, VI
Blair (2000)^45^	124	37	61	Cambodia	USA	I, V
Gernaat et al (2002)^46^	51	38	47	Afghanistan	The Netherlands	I, III, V
Lie (2002)^47^	240	41	51	Mix	Norway	VI
Rothe et al (2002)^48^	87	15	43	Cuba	USA	VI
Slodnjak et al (2002)^49^	265	15	53	Bosnia and Herzegovina	Slovenia	II, VI
Keller et al (2003)^50^	70	28	20	Mix	USA	II, IV, VI
Turner et al (2003)^51^	120	38	53	Kosovo	UK	IV
Fox et al (2004)^52^	237	11	54	Mix	USA	IV
Jaranson et al (2004)^53^	1134	35	47	Mix	USA	VI
Lie (2004)^54^	175	43	47	Bosnia and Herzegovina	Norway	II, IV, VI
Laban et al (2005)^55^	294	35	35	Iraq	The Netherlands	I, III, V
Marshall et al (2005)^56^	490	52	65	Cambodia	USA	I, V
Steel et al (2005)^57^	1161	39	35	Vietnam	Australia	I, III, V
Bhui et al (2006)^58^	143	35	50	Somalia	UK	I, III, V
Roth & Ekblad (2006)^59^	91	42	40	Kosovo	Sweden	II
Schweitzer et al (2006)^60^	63	34	33	Sudan	Australia	II, VI
Ahmad et al (2008)^61^	290	30	57	Mix	Sweden	VI
Hodes et al (2008)^62^	109	17	39	Mix	UK	VI
Coffey et al (2010)^63^	17	42	94	Mix	Australia	II, VI
Nickerson et al (2010)^64^	315	38	52	Iraq	Australia	II, VI
Silove et al (2010)^65^	126	47	61	Bosnia and Herzegovina	Australia	I, III, V
Beiser et al (2011)^66^	1603	43	46	Sri Lanka	Canada	V
Groark et al (2011)^67^	6	17	33	Mix	UK	I, III, V
Muhtz et al (2011)^68^	502	71	56	Eastern Germany	Germany	V
Bogic et al (2012)^69^	854	42	51	Yugoslavia	Mix	I, III, V
Heeren et al (2012)^70^	86	30	65	Mix	Switzerland	I, III, V
Rasmussen et al (2012)^71^	660	47	48	Mix	USA	I, III, V
Warfa et al (2012)^72^	189	34	48	Somalia	Mix	I, III, V
Bronstein et al (2013)^73^	222	16	0	Afghanistan	UK	II, IV
Cleveland & Rousseau (2013)^74^	188	33	40	Mix	Canada	II, IV, VI
Hollifield et al (2013)^75^	251	33	50	Mix	USA	II, IV, VI
Rees et al (2013)^76^	44	39	16	Papua-New-Guinea	Australia	VI
Tay et al (2013)^77^	52	39	35	Mix	Australia	III, V
Heeren et al (2014)^78^	99	34	49	Mix	Switzerland	II, IV, IV
Lamkaddem et al (2014)^79^	172	39	51	Mix	The Netherlands	VI
Mölsä et al (2014)^80^	128	59	60	Somalia	Finland	II
Slewa-Younan et al (2014)^81^	225	38	56	Iraq	Australia	VI
Vervliet et al (2014)^82^	77	16	13	Mix	Belgium	II, IV, VI
Völkl-Kernstock et al (2014)^83^	41	17	15	Mix	Austria	VI
Hocking & Sundram (2015)^84^	131	35	16	Mix	Australia	II, VI
Jensen et al (2015)^85^	93	14	19	Somalia Mix	Norway	II, IV, VI
McGregor et al (2015)^86^	10	18	80	Mix	Australia	VI
Vonnahme et al (2015)^87^	386	34	47	Bhutan	USA	II, IV, VI
Morina et al (2016)^88^	51	43	45	Mix	Switzerland	II, IV, VI
Park et al (2017)^89^	131	19	65	North-Korea	South-Korea	II
Georgiadou et al (2018)^90^	200	33	31	Syria	Germany	II, VI, V
Javanbakht et al (2018)^91^	167	47	50	Syria	USA	II, IV, VI
Richter et al (2018)^92^	56	32	44	Mix	Germany	I, V
Schweitzer et al (2018)^93^	104	32	100	Mix	Australia	II, IV, VI
Kartal et al (2019)^94^	138	40	45	Bosnia	Australia, Austria	II, IV, VI
Leiler et al (2019)^95^	367	30	27	Mix	Sweden	II, IV, VI
Poudel-Tandukar et al (2019)^96^	225	39	50	Bhutan	USA	II, IV

a. Where the mean age of the sample was not available we report the median age of the sample.

b. This column indicates in which meta-analysis the study is included: I, depression diagnosis; II, depression self-report; III, anxiety diagnosis; IV, anxiety self-report; V, post-traumatic stress disorder (PTSD) diagnosis, VI, PTSD self-report.

#### Quality assessment

Methodological quality scores for the included studies ranged between −1.5 and 9 (mean 4.3, s.d. = 2.5; supplementary Tables 2 and 3). The interrater reliability of the methodological quality assessments was high (*κ* = 0.79, s.e. = 0.09).^97^ On average, the methodological quality score of the included studies was modest to good. Most studies were clear in the formulation of study goals, population and participation rate. However, hardly any study assessed potential confounding variables or performed follow-up assessments. Obviously, no studies were masked (‘blinded’) to participant status.

#### Prevalence of anxiety, depression and PTSD in adult and child/adolescent refugees

Table 2 provides overall random-effects pooled prevalence estimates for anxiety, depression and PTSD in refugees by assessment method (i.e. self-report versus diagnostic interview) and by age status (i.e. child/adolescent versus adult). For forest plots on these estimates we refer to supplementary Figs 1–6. Prevalence estimates were on average higher when they were derived from self-report rather than interview. This difference was statistically significant for anxiety disorders, where a 29% difference in prevalence rates was observed. The differences in prevalence estimates as a function of assessment method for depression (10%) and PTSD (8%) were not significant. Prevalence estimates were high in both child/adolescent and adult samples, with no statistically significant differences between the age groups. Between-study heterogeneity was high in all analyses. Supplementary Table 4 presents prevalence estimates by child/adolescent and adult refugee samples and assessment method (i.e. self-report versus diagnostic interview).

**Table 2:** Prevalence of anxiety, depression and post-traumatic stress disorder by assessment method

	*k*^a^	*n*^a^	Prevalence (95% CI)	*I*^2^	Kendall's tau^b^
Anxiety	36	6728	0.30 (0.22–0.37)	99.1***	0.37*
Diagnosis	16	3634	0.13 (0.08–0.17)^c^	95.4***	
Self-report	20	3094	0.42 (0.31–0.52)^c^	98.2***	
Children/adolescents	5	493	0.32 (0.28–0.37)	98.6***	
Adults	28	5911	0.28 (0.19–0.38)	98.2***	
Depression	55	10 466	0.36 (0.30–0.42)	98.6***	0.13
Diagnosis	23	5230	0.30 (0.23–0.38)	98.0***	
Self-report	32	5236	0.40 (0.31–0.48)	98.3***	
Children/adolescents	7	995	0.28 (0.19–0.37)	90.4***	
Adults	40	8750	0.36 (0.30–0.43)	98.6***	
PTSD	59	13 288	0.34 (0.29–0.40)	99.1***	0.14
Diagnosis	26	7578	0.29 (0.22–0.37)	99.1***	
Self-report	33	5710	0.37 (0.30–0.45)	98.0***	
Children/adolescents	7	662	0.52 (0.35–0.68)	94.5***	
Adults	42	11 948	0.29 (0.23–0.36)	99.2***	

a. Numbers for *k* (prevalence estimates per analysis) and *n* (number of subjects per analysis) do not add up to the total in pooled estimates reported separately for mixed child/adolescent and adult refugees. This is due to the inclusion of some samples that assessed mental health in mixed child/adolescent and adult refugee groups in our study and these could not be categorised in a single age category.

b. Kendall's tau: rank correlation test for funnel-plot asymmetry. A significant correlation is an indication of the presence of publication bias.

c. Difference in proportions: *Z* = −1.96, *P* < 0.05.

*P < 0.05; **P < 0.01; ***P < 0.001.

In supplementary Table 5, prevalence rates of anxiety, depression and PTSD in child/adolescent and adult refugees are set out against rates in non-refugee populations living in conflict or war settings. Prevalence rates for all three disorders are substantially higher in refugees relative to those reported in non-refugees over the globe and this is so for both child/adolescent and adult refugees (all *P* < 0.05). In adult refugees, prevalence rates of anxiety, depression and PTSD are significantly higher than in populations living in conflict or war settings. This latter difference was not statistically significant in child/adolescent refugees.

#### Moderator analysis

Prevalence rates of anxiety, depression and PTSD did not differ as a function of continent of origin or continent of resettlement (supplementary Tables 6 and 7). Differences in prevalence rates based on the years that the input studies were published were not observed (supplementary Table 8). Prevalence rates were also not associated with the average duration of residence, mean age and gender distribution of the sample, nor with the methodological quality of the study (supplementary Table 9). Supplementary Table 10 provides information on the associations among the moderators. In about 10% of the included articles it was not clear whether language-adapted assessments were performed or whether an interpreter was present during the assessment. The prevalence rates reported in these studies did not differ significantly from those reported in articles in which it was clear whether language adaptation was applied or an interpreter was present.

## Discussion

This systematic review with meta-analyses shows that up to 1 in 3 refugees has diagnosable current depression and/or PTSD. Diagnosable anxiety disorders are estimated to be present in 1–2 out of 10 refugees. The prevalence of these disorders assessed by cut-off scores on self-report instruments is even higher. Together these findings, evidentially, suggest a significant and chronic burden in refugees due to poor mental health, impeding their functioning and possibilities to adapt.^1,98^

When method of assessment is considered, the results reported here are largely in line with the results reported in earlier meta-analyses.^14–16^ This could suggest that prevalence rates of anxiety, depression and PTSD in refugee populations do not change over time. Strengthening this suggestion is that we did not find evidence that prevalence rates depended on the year that the input studies were published.

### Risk factors for mental disorders in refugee populations

The pooled prevalence rates we report resemble those for other traumatised populations (e.g. childhood sexual or emotional abuse), with particularly strong associations with PTSD and depression, and moderate associations with anxiety.^99–101^ Prevalence rates of anxiety, depression and PTSD among adult refugees are high relative not only to non-refugee populations, but also to populations living in conflict or war settings. This seems to suggest that it is not only the exposure to conflict and war itself that makes a refugee vulnerable to, for instance, PTSD, but that the flight and/or additional post-migration factors may aggravate the trauma-related symptoms. For anxiety disorders and PTSD, we found similar trends for child/adolescent refugees, although these were not statistically significant. As only five papers were included on childhood/adolescent anxiety and seven on childhood/adolescent depression and PTSD, the lack of significance could well be due to insufficient statistical power.

Besides pre-migration factors such as exposure to war, torture or persecution, post-migration factors, including life-threatening journeys, long-lasting asylum procedures, family separation, unemployment and discrimination, have consistently been shown to affect prevalence of mental disorders.^6,94,102^ Awareness of the role of post-migration stressors needs to increase since, unlike pre-migration stressors, policy makers and clinicians may have the power to change them. Clinically, it is highly relevant to elucidate in more detail which pre-, peri- and post-migration factors specifically contribute to the depression, anxiety and PTSD symptoms. Follow-up studies have directly compared refugees from one country or region with individuals who stayed in that area, considering individual and environmental risk and resilience factors, such as types of traumatic and stressful life event, personality characteristics, socioeconomic status and resources. Such knowledge may help in the development of prevention strategies and scalable treatment options that specifically could help those refugees in need of care.^103^

In the current study we had only limited information on such pre- and post-migration factors. We tried to cluster reasons for fleeing, for example war or violence versus natural disasters. This attempt was unsuccessful because of heterogeneity and a lack of clear information in articles. The only variable linking to post-migration factors that was consistently reported over studies was length of residence. Remarkably, length of residence was found to be unrelated to prevalence rates. This seems to indicate that time in itself does not have much of a healing effect. However, this finding should be viewed with caution. First, the potential association between length of residence and prevalence of mental disorders was assessed by means of meta-regression and this may have been underpowered owing to the small number of observations and the use of study averages.^104^ Second, length of residence probably interacts with other post-migration factors (e.g. whether permanent residence is received) in the outcomes of our meta-analyses.

The current research reports high prevalence of anxiety, depression and PTSD for both male and female adult and child/adolescent refugees. We did not observed moderating effects of mean age and gender distribution of a sample on prevalence rates, despite previous meta-analytic findings and reviews showing indications for differences in prevalence rates as a function of age.^9,10^ For instance, the meta-analysis by Fazel et al^14^ showed higher prevalence of PTSD for adolescents and young adults compared with adults. These differences are also evident in our study, as PTSD prevalence rates are reported to be 0.27 in adults and 0.52 in children/adolescents, yet with overlapping confidence intervals.

### Assessment by self-report versus diagnostic interview

Self-report screening instruments are popular in the assessment of refugees because they are widely available in many languages, are easy to administer and incur low costs. Earlier meta-analyses in this field excluding studies that used self-report screening instruments^14^ featured only a quarter of the data compared with studies^15^ that included publications based on both assessment types. The main difference between these instruments is that self-report measures at best yield caseness of a mental disorder, whereas interviews yield a formal diagnosis. The latter is stricter, since the core symptoms of a disease and significant interference with everyday life need to be present for formal diagnosis, whereas this is not necessary for caseness. This may explain the higher prevalence rates when assessments were based on self-report. We found the difference in prevalence rate as a function of assessment method to be statistically significant only for anxiety disorders. Perhaps this could be due to the large overlap between anxiety and PTSD and their diagnostic clustering in previous diagnostic systems (e.g. DSM-IV-TR). This might have resulted in the development of self-report screening instruments for anxiety that potentially capture a mix of anxiety and stress-related constructs, whereas (subtle) distinctions between the two could be made in a clinical interview.

It is important to investigate whether the course of illness and adjustment to the new home situation is different for refugees with diagnosed disorders compared with those who score above a cut-off score on a self-report questionnaire. Likewise, it would be interesting to investigate whether these groups differ from each other with regard to peri- and pre-migration characteristics and events.

### Limitations and strengths

There are several limitations to this study, besides the above-mentioned power and measurement problems. The general refugee population is an extremely heterogeneous population, difficult to assess for research purposes, and therefore many studies have to rely on small samples and non-random sampling methods.^7,58^ A large body of research in this field includes only samples assessed in high-income countries. As our selection method excluded other data, generalisation of our results to refugees in lower-income countries is limited. Furthermore, this study does not focus on the burden of displacement within a country. However, since lower-income countries see the largest influx of refugees in connection to a war or other crises in a neighbouring country,^2,105^ and most refugees worldwide reside in lower-income countries,^10^ more research on these samples is needed. Much of the between-study heterogeneity in our analyses remained unexplained. Post-migration factors such as migrant status, socioeconomic position and family reunification are projected to explain parts of this heterogeneity. Unfortunately, detailed information on these variables was not available in most of the included studies, so we could not formally test the impact of these factors.

The assessment of several moderators and our broad approach followed by sensitivity analyses (e.g. we included two assessment methods and then conducted stratified analyses) yielded additional insight into the prevalence of anxiety, depression and PTSD in the refugee populations. So, we consider this as a strong point of our study.

### Research and clinical implications

The World Health Organization has recently stated that prevalence of mental disorders in refugees is an important factor for consideration in developing effective policies.^1,2^ Our data show that refugees are highly vulnerable to mental disorders even years after resettling in a high-income country. This is important and alarming in itself, but even more so considering the increasing growth in numbers of refugees across the globe. On the basis of our findings we advocate for more research on prevention, and support further development of scalable treatments for this heterogeneous high-risk population.

## Data Availability

The data that support the findings of this study are available from the corresponding author on reasonable request.

## Supplementary material

For supplementary material accompanying this paper visit http://dx.doi.org/10.1192/bjo.2020.54. click here to view supplementary material
