# Supplementary material for: Anxiety, depression and post-traumatic stress disorder in refugees resettling in high-income countries: systematic review and meta-analysis
Source: BJPsych Open. 2020 Jul 2;6(4):e68. doi: 10.1192/bjo.2020.54 (PMC7443922; doi:10.1192/bjo.2020.54)
Supplement: Supplementary file 1 [file S205647242000054Xsup001.zip › S205647242000054Xsup001.docx]

**Supplement: Anxiety, depression and post-traumatic stress disorder in refugees resettling in developed countries: a systematic review and meta-analysis**

-- Henkelmann *et al.*

**Table S1**. Characteristics of included studies and samples.

| **Study** | **Diagnostic Instrument** | | **Outcome** | **Duration of stay (mos)** |
| --- | --- | --- | --- | --- |
| Westermeyer (1988) | Diagnostic interview scale | | Diagnosis | 84 |
| Hinton *et al.* (1993) | Structured clinical interview for DSM-III- R, Anxiety disorder interview schedule revised | | Diagnosis | 3 median (range 0-6) |
| Carlson and Rosser-Hogan (1994) | Hopkins symptom checklist-25, PTSD checklist | | Self-Report | 60 |
| Cheung (1994) | Diagnostic interview scale | | Diagnosis | 62 median (range 3-120) |
| Pernice and Brook (1994) | Hopkins symptom checklist-25 | | Self-Report | *N.K.* |
| Weine *et al.* (1995) | PTSD symptom scale, Symptom checklist-90 | | Self-Report | *N.K.* |
| Malekzai *et al.* (1996) | Clinician administered PTSD scale for DSM-III | | Diagnosis | 60 median (range 0 – 120) |
| D’Avanzo and Barab (1998) | Hopkins symptom checklist-25 | | Self-Report | min 60 |
| Almqvist and Broberg (1999) | Impact of event scale | | Self-Report | 42 |
| Favaro *et al.* (1999) | Structured clinical interview for DSM-IV | | Diagnosis | 42 |
| Mollica *et al.* (1999) | Hopkins symptom checklist-25, Harvard trauma questionnaire | | Self-Report | 36 |
| Sack *et al.* (1999) | Schedule for affective disorders and schizophrenia for school-aged children, Diagnostic interview for children/adolescents | | Diagnosis | 66 |
| Tousignant *et al.* (1999) | Diagnostic interview schedule for children | | Diagnosis | 36 minimal |
| Papageorgiou *et al.* (2000) | Revised children’s manifest anxiety scale, Impact of event scale | | Self-Report | *N.K.* |
| Gernaat *et al.* (2002) | Composite international diagnostic interview | | Diagnosis | 46 |
| Lie (2002) | PTSD for children questionnaire | | Self-Report | 46 |
| Rothe *et al.* (2002) | PTST reactive index | | Self-Report | 12 |
| Slodnjak *et al.* (2002) | Depression self-rating scale for children, Impact of event scale | | Self-Report | *N.K.* |
| Keller *et al.* (2003) | Hopkins symptom checklist-25, Harvard trauma questionnaire | | Self-Report | 7 |
| Turner *et al.* (2003) | Beck’s anxiety and depression inventory | | Self-Report | *N.K.* |
| Fox *et al.* (2004) | Depression self-rating scale for children | | Self-Report | *N.K.* |
| Jaranson *et al.* (2004) | PTSD checklist for DSM civilian version | | Self-Report | 41 |
| Lie (2004) | Hopkins symptom checklist-25, Harvard trauma questionnaire | | Self-Report | *N.K.* |
| Laban *et al.* (2005) | Composite international diagnostic interview | | Diagnosis | 20 |
| Marshall *et al.* (2005) | Composite international diagnostic interview | | Diagnosis | 252 |
| Steel *et al.* (2005) | Composite international diagnostic interview | | Diagnosis | 136 |
| Bhui *et al.* (2006) | Mini International neuropsychiatric interview, Composite international diagnostic interview | | Diagnosis | 33 median (range 0-66) |
| ***Table S1*** *continues on the next page* | |  |  |  |
| ***Table S1*** *continued* |  | |  |  |
| Roth and Ekblad (2006) | Hopkins symptom checklist-25 | | Self-Report | 6 |
| Schweitzer *et al.* (2006) | Hopkins symptom checklist-37, Harvard trauma questionnaire | | Self-Report | 144 median (range 0-288) |
| Ahmad *et al.* (2008) | Hopkins symptom checklist-25, PTSD questionnaire for children | | Self-Report | 99.6 |
| Hodes *et al.* (2008) | Depression self-rating scale for children, Impact of event scale | | Self-Report | 31 |
| Coffey *et al.* (2010) | Hopkins symptom checklist-25, Harvard trauma questionnaire | | Self-Report | 83 |
| Nickerson *et al.* (2010) | Hopkins symptom checklist-25, Harvard trauma questionnaire | | Self-Report | 56 |
| Silove *et al.* (2010) | Structured clinical interview for DSM-IV, CAPS | | Diagnosis | 36 minimal |
| Beiser *et al.* (2011) | PTSD checklist | | Diagnosis | 118 |
| Groark *et al.* (2011) | Spence child anxiety scale, Depression self-rating scale for children, Impact of event scale | | Diagnosis | 9 |
| Muhtz *et al.* (2011) | Structured clinical interview for DSM-IV | | Diagnosis | 720 |
| Blair (2012) | Diagnostic interview scale for children/adolescents | | Diagnosis | 97 |
| Bogic *et al.* (2012) | Mini international neuropsychiatric interview | | Diagnosis | 112 |
| Heeren *et al.* (2012) | Mini international neuropsychiatric interview | | Diagnosis | 3 |
| Heeren *et al.* (2012) | Mini international neuropsychiatric interview | | Diagnosis | 16 |
| Rasmussen *et al.* (2012) | Composite international diagnostic interview | | Diagnosis | 236 |
| Warfa *et al.* (2012) | Mini international neuropsychiatric interview | | Diagnosis | *N.K.* |
| Bronstein *et al.* (2013) | Hopkins symptom checklist-37A | | Self-Report | 19 |
| Cleveland and Rousseau (2013) | Hopkins symptom checklist-25, Harvard trauma questionnaire | | Self-Report | 3 |
| Cleveland and Rousseau (2013) | Hopkins symptom checklist-25, Harvard trauma questionnaire | | Self-Report | 1 detained |
| Hollifield *et al.* (2013) | Hopkins symptom checklist-25, PTSD symptom scale | | Self-Report | *N.K.* |
| Rees *et al.* (2013) | Harvard trauma questionnaire | | Self-Report | 231 |
| Tay *et al.* (2013) | Structured clinical interview for DSM-IV | | Diagnosis | 5 |
| Heeren *et al.* (2014) | Hopkins symptom checklist-25, Harvard trauma questionnaire, Posttraumatic diagnostic scale | | Self-Report | 66 |
| Lamkaddem *et al.* (2014) | Harvard trauma questionnaire | | Self-Report | 84 |
| Mölsä *et al.* (2014) | Beck’s depression inventory-21 | | Self-Report | 114 |
| Slewa-Younan *et al.* (2014) | Harvard trauma questionnaire | | Self-Report | 59 |
| Vervliet *et al.* (2014) | Hopkins symptom checklist-37A, Children’s revised impact of event scale | | Self-Report | 18 |
| Völkl-Kernstock *et al.* (2014) | PTSD reaction index | | Self-Report | *N.K.* |
| ***S1*** *continues on the next page* | | | | |
| ***Table S1*** *continued* |  | |  |  |
| Hocking and Sundram (2015) | Hopkins symptom checklist-25, Harvard trauma questionnaire | | Self-Report | *N.K.* |
| Jensen *et al.* (2015) | Composite international diagnostic interview, Hopkins symptom checklist 25, CPSS | | Self-Report | 6 |
| McGregor *et al.* (2015) | Child PTSD symptom scale | | Self-Report | 78 |
| Vonnahme *et al.* (2015) | Hopkins symptom checklist-25, Harvard trauma questionnaire | | Self-Report | 20 |
| Morina *et al.* (2016) | Hopkins symptom checklist-25, Posttraumatic diagnostic scale | | Self-Report | 136 |
| Park *et al.* (2017) | Children’s depression inventory | | Self-Report | *N.K.* |
| Georgiadou *et al.* (2018) | Patient health questionnaire-9, Essen trauma inventory | | Self-Report | 23 |
| Javanbakht *et al.* (2018a) | PTSD checklist civilian version, Hopkins symptom checklist-25 | | Self-Report | *N.K.* |
| Javanbakht *et al.* (2018b) | PTSD checklist civilian version, Hopkins symptom checklist-25 | | Self-Report | *N.K.* |
| Richter *et al.* (2018) | Mini international neuropsychiatric interview | | Diagnosis | *N.K.* |
| Schweitzer *et al.* (2018) | Hopkins symptom checklist-25, Harvard trauma questionnaire | | Self-Report | 3 |
| Kartal et al. (2019) | Harvard trauma questionnaire, Depression anxiety stress scale, Posttraumatic stress diagnostic scale | | Self-Report | 212 |
| Leiler *et al.* (2019) | Patient health questionnaire-9, Generalized anxiety disorder 7, Primary care PTSD screen | | Self-Report | *N.K.* |
| Poudel-Tandukar *et al.* (2019) | Hopkins symptom checklist-25 | | Self-Report | 48 |

**Methodological quality assessment**

The *quality assessment tool for cross-sectional studies* (United States National Institutes of Health; https://www.nhlbi.nih.gov/health-topics/study-quality-assessment-tools) was used to assess the methodological quality of the included studies. Three items of this tool were not relevant for the papers in the current systematic review and meta analyses, and hence they were excluded (see **Table S2** for the excluded items and the corresponding reasons for exclusion).

**Table S2** Excluded assessment items, with reason. For the full list of items, we refer to the U.S. NIH: https://www.nhlbi.nih.gov/health-topics/study-quality-assessment-tools.

| **Item** | **Reason for exclusion** |
| --- | --- |
| Exposure(s) assessed more than once over time | Exposure is refugee status |
| Outcome assessors blinded to exposure status of participants | Blinding not possible (*i.e.,* self-report, interview) |
| Loss to follow-up after baseline 20% or less | Longitudinal nature was not relevant for inclusion |

Quality assessments were performed independently by two members of the review team (CD and MM). The overall agreement with regard to quality assessment was high (Kappa [κ] = 0.79, *SE* = 0.04, *P* <. 001). Average methodological quality score of the included studies was 4.2 (range = -2 to 9). The methodological quality score was related to the main outcome measures of our study in meta-regression analyses and it was not used for decisions about in- or exclusion of a study in our meta-analyses. **Table S3** provides the item and total scores per included study as scored by both reviewers.

**Table S3** Quality assessment by study and reviewer. Studies are presented chronologically, and alphabetically within year.

| **Study** | **1** | **2** | **3** | **4** | **5** | **6** | **7** | **8** | **9** | **11A** | **11B** | **12** | **13** | **15** | **Total** |
| --- | --- | --- | --- | --- | --- | --- | --- | --- | --- | --- | --- | --- | --- | --- | --- |
| Westermeyer (1988) C.D. | **⊕** | **⊕** | **⊕** | **⊕** | **∅** | **⊕** | **⊕** | **∅** | **⊕** | **⊕** | **⊕** | **∅** | **⊗** | **⊗** | 6 |
| Westermeyer (1988) M.M. | **∅** | **⊕** | **⊕** | **⊕** | **∅** | **⊕** | **⊕** | **∅** | **⊕** | **⊕** | **⊕** | **∅** | **⊗** | **⊗** | 5 |
| Hinton *et al.* (1993) C.D. | **⊕** | **⊕** | **⊕** | **⊕** | **∅** | **⊕** | **⊕** | **∅** | **⊕** | **⊕** | **⊕** | **∅** | **⊗** | **⊕** | 9 |
| Hinton *et al.* (1993) M.M. | **⊕** | **⊕** | **⊕** | **⊕** | **∅** | **⊕** | **⊕** | **∅** | **⊕** | **⊕** | **⊕** | **∅** | **⊗** | **⊕** | 9 |
| Carlon & Roser-Hogan (1994) C.D. | **⊕** | **⊕** | **⊕** | **⊕** | **∅** | **⊕** | **⊕** | **∅** | **⊕** | **⊗** | **⊕** | **∅** | **⊗** | **⊗** | 5 |
| Carlon & Rossr-Hogan (1994) M.M. | **⊕** | **⊕** | **⊕** | **⊕** | **∅** | **⊕** | **⊕** | **∅** | **⊕** | **⊗** | **⊕** | **∅** | **⊗** | **⊗** | 5 |
| Cheung (1994) C.D. | **⊕** | **⊕** | **∅** | **⊕** | **∅** | **⊕** | **⊕** | **∅** | **∅** | **⊕** | **⊕** | **∅** | **⊗** | **⊕** | 7 |
| Cheung (1994) M.M. | **⊕** | **⊕** | **∅** | **⊕** | **∅** | **⊕** | **⊕** | **∅** | **∅** | **⊕** | **⊕** | **∅** | **∅** | **∅** | 7 |
| Pernice & Brook (1994) C.D. | **⊕** | **⊕** | **∅** | **⊕** | **∅** | **⊕** | **⊕** | **∅** | **⊕** | **⊗** | **⊕** | **∅** | **⊗** | **⊗** | 4 |
| Pernice & Brook (1994) M.M. | **⊕** | **⊗** | **∅** | **⊕** | **∅** | **⊕** | **⊕** | **∅** | **⊕** | **⊗** | **⊕** | **∅** | **⊗** | **⊗** | 2 |
| Weine *et al.* (1995) C.D. | **⊕** | **⊕** | **⊕** | **⊕** | **∅** | **⊕** | **⊕** | **∅** | **⊕** | **⊗** | **⊕** | **∅** | **⊗** | **⊗** | 5 |
| Weine *et al.* (1995) M.M. | **⊕** | **⊕** | **⊕** | **⊗** | **∅** | **⊕** | **⊕** | **∅** | **⊕** | **⊗** | **⊕** | **∅** | **⊗** | **⊗** | 3 |
| Malekzai *et al.* (1996) C.D. | **⊕** | **⊕** | **⊗** | **⊗** | **∅** | **⊕** | **⊕** | **∅** | **⊕** | **⊕** | **⊕** | **∅** | **⊗** | **⊗** | 3 |
| Malekzai *et al.* (1996) M.M. | **∅** | **∅** | **⊗** | **⊗** | **∅** | **⊕** | **⊕** | **∅** | **⊕** | **⊕** | **⊕** | **∅** | **⊗** | **⊗** | 1 |
| D’Avanzo & Barab (1998) C.D. | **⊕** | **⊕** | **∅** | **⊕** | **∅** | **⊕** | **⊕** | **∅** | **⊕** | **⊗** | **⊕** | **∅** | **⊗** | **⊗** | 4 |
| D’Avanzo & Barab (1998) M.M. | **⊕** | **⊕** | **∅** | **⊕** | **∅** | **⊕** | **⊕** | **∅** | **∅** | **⊗** | **⊕** | **∅** | **⊗** | **⊗** | 3 |
| Almqvist & Broberg (1999) C.D. | **⊕** | **⊕** | **⊕** | **⊕** | **∅** | **⊕** | **⊕** | **∅** | **⊕** | **⊗** | **⊕** | **∅** | **⊕** | **⊕** | 9 |
| Almqvist & Broberg (1999) M.M. | **⊕** | **⊕** | **⊕** | **⊕** | **∅** | **⊕** | **⊕** | **∅** | **⊕** | **⊗** | **⊕** | **∅** | **⊕** | **⊕** | 9 |
| Favaro *et al.* (1999) C.D. | **⊕** | **⊕** | **∅** | **⊕** | **∅** | **⊕** | **⊕** | **∅** | **⊕** | **⊗** | **⊕** | **∅** | **⊗** | **⊗** | 4 |
| Favaro *et al.* (1999) M.M. | **⊕** | **⊕** | **∅** | **⊕** | **∅** | **⊕** | **⊕** | **∅** | **⊕** | **⊗** | **⊕** | **∅** | **⊗** | **⊗** | 4 |
| Mollica *et al.* (1999) C.D. | **⊕** | **⊕** | **⊕** | **⊕** | **∅** | **⊕** | **⊕** | **∅** | **⊕** | **⊗** | **⊕** | **∅** | **⊗** | **⊕** | 7 |
| Mollica *et al.* (1999) M.M. | **⊕** | **⊕** | **⊕** | **⊕** | **∅** | **⊕** | **⊕** | **∅** | **⊕** | **⊗** | **⊕** | **∅** | **⊗** | **⊕** | 7 |
| Sack *et al.* (1999) C.D. | **⊕** | **⊕** | **⊕** | **⊕** | **∅** | **⊕** | **⊕** | **∅** | **⊗** | **⊕** | **⊕** | **∅** | **⊕** | **⊗** | 7 |
| Sack *et al.* (1999) M.M. | **∅** | **∅** | **⊕** | **⊕** | **∅** | **⊕** | **⊕** | **∅** | **⊗** | **⊕** | **⊕** | **∅** | **⊕** | **⊗** | 5 |
| Tousignant *et al.* (1999) C.D. | **⊕** | **⊕** | **⊕** | **⊗** | **∅** | **⊕** | **⊕** | **∅** | **⊗** | **⊕** | **⊕** | **∅** | **⊗** | **⊗** | 3 |
| ***Table S3*** *continues on the next page* | | | | | | | | | | | | | | | |
| ***Table S3*** *continued* | | | | | | | | | | | | | | | |
| Tousignant *et al.* (1999) M.M. | **⊕** | **⊕** | **⊕** | **⊗** | **∅** | **⊕** | **⊕** | **∅** | **⊗** | **⊕** | **⊕** | **∅** | **⊗** | **⊗** | 3 |
| Papageorgiou *et al.* (2000) C.D. | **⊕** | **⊕** | **⊕** | **⊗** | **∅** | **⊕** | **⊕** | **∅** | **⊗** | **⊗** | **⊕** | **∅** | **⊗** | **⊗** | 1 |
| Papageorgiou *et al.* (2000) M.M. | **⊕** | **⊕** | **⊕** | **⊗** | **∅** | **⊕** | **⊕** | **∅** | **⊗** | **⊗** | **⊕** | **∅** | **∅** | **∅** | 3 |
| Gernaat *et al.* (2002) C.D. | **⊕** | **⊕** | **⊕** | **⊕** | **∅** | **⊕** | **⊕** | **∅** | **⊗** | **⊕** | **⊕** | **∅** | **⊕** | **⊗** | 7 |
| Gernaat *et al.* (2002) M.M. | **⊕** | **∅** | **⊕** | **⊕** | **∅** | **⊕** | **⊕** | **∅** | **⊗** | **⊕** | **⊕** | **∅** | **⊕** | **⊗** | 6 |
| Lie (2002) C.D. | **⊕** | **⊕** | **⊕** | **⊕** | **∅** | **⊕** | **⊕** | **∅** | **⊕** | **⊗** | **⊕** | **∅** | **⊕** | **⊕** | 9 |
| Lie (2002) M.M. | **⊕** | **⊕** | **⊕** | **∅** | **∅** | **⊕** | **⊕** | **∅** | **⊕** | **⊗** | **⊕** | **∅** | **⊕** | **∅** | 7 |
| Rothe *et al.* (2002) C.D. | **⊕** | **⊕** | **⊕** | **⊕** | **∅** | **⊕** | **⊕** | **∅** | **⊗** | **⊗** | **⊕** | **∅** | **⊗** | **⊕** | 5 |
| Rothe *et al.* (2002) M.M. | **⊕** | **⊕** | **⊕** | **⊕** | **∅** | **⊕** | **⊕** | **∅** | **⊗** | **∅** | **⊕** | **∅** | **⊗** | **⊕** | 4 |
| Slodnjak *et al.* (2002) C.D. | **⊕** | **⊕** | **⊗** | **⊗** | **∅** | **⊕** | **⊕** | **∅** | **⊗** | **⊗** | **⊕** | **∅** | **⊗** | **⊗** | -1 |
| Slodnjak *et al.* (2002) M.M. | **⊕** | **⊕** | **⊗** | **⊗** | **∅** | **⊕** | **⊕** | **∅** | **⊗** | **⊗** | **⊕** | **∅** | **⊗** | **⊗** | -1 |
| Keller *et al.* (2003) C.D. | **⊕** | **⊕** | **∅** | **∅** | **∅** | **⊕** | **⊕** | **∅** | **⊗** | **⊗** | **⊕** | **∅** | **⊕** | **⊗** | 3 |
| Keller *et al.* (2003) M.M. | **⊕** | **⊕** | **∅** | **∅** | **∅** | **⊕** | **⊕** | **∅** | **⊗** | **⊗** | **⊕** | **∅** | **⊕** | **⊗** | 3 |
| Turner *et al.* (2003) C.D. | **⊕** | **∅** | **∅** | **∅** | **∅** | **⊕** | **⊕** | **∅** | **⊗** | **⊗** | **⊕** | **∅** | **∅** | **⊗** | -1 |
| Turner *et al.* (2003) M.M. | **⊕** | **∅** | **∅** | **∅** | **∅** | **⊕** | **⊕** | **∅** | **⊗** | **⊗** | **⊕** | **∅** | **⊗** | **⊗** | 0 |
| Fox *et al.* (2004) C.D. | **⊕** | **⊕** | **∅** | **⊕** | **∅** | **⊕** | **⊕** | **∅** | **⊗** | **⊗** | **⊕** | **∅** | **⊗** | **⊗** | 2 |
| Fox *et al.* (2004) M.M. | **⊕** | **⊕** | **∅** | **⊕** | **∅** | **⊕** | **⊕** | **∅** | **⊗** | **⊗** | **⊕** | **∅** | **⊗** | **⊗** | 2 |
| Jaranson *et al.* (2004) C.D. | **⊕** | **⊕** | **⊕** | **⊗** | **∅** | **⊕** | **⊕** | **∅** | **⊗** | **⊗** | **⊕** | **∅** | **⊗** | **⊕** | 3 |
| Jaranson *et al.* (2004) M.M. | **⊕** | **⊕** | **⊕** | **⊗** | **∅** | **⊕** | **⊕** | **∅** | **∅** | **⊗** | **⊕** | **∅** | **∅** | **⊕** | 1 |
| Lie (2004) C.D. | **⊕** | **⊕** | **⊕** | **⊕** | **∅** | **⊕** | **⊕** | **∅** | **⊗** | **⊗** | **⊕** | **∅** | **⊕** | **⊕** | 7 |
| Lie (2004) M.M. | **⊕** | **⊕** | **⊕** | **⊕** | **∅** | **⊕** | **⊕** | **∅** | **⊗** | **∅** | **∅** | **∅** | **⊕** | **⊕** | 5 |
| Laban *et al.* (2005) C.D. | **⊕** | **⊕** | **⊕** | **⊕** | **∅** | **⊕** | **⊕** | **∅** | **⊗** | **⊕** | **⊕** | **∅** | **⊗** | **⊕** | 8 |
| Laban *et al.* (2005) M.M. | **⊕** | **⊕** | **⊕** | **⊕** | **∅** | **⊕** | **⊕** | **∅** | **⊗** | **⊕** | **⊕** | **∅** | **⊗** | **⊕** | 8 |
| Marshall *et al.* (2005) C.D. | **⊕** | **⊕** | **⊕** | **⊕** | **∅** | **⊕** | **⊕** | **∅** | **⊕** | **⊕** | **⊕** | **∅** | **⊗** | **⊕** | 9 |
| Marshall *et al.* (2005) M.M. | **⊕** | **⊕** | **⊕** | **⊕** | **∅** | **⊕** | **⊕** | **∅** | **∅** | **⊕** | **⊕** | **∅** | **⊗** | **⊕** | 8 |
| Steel *et al.* (2005) C.D. | **⊕** | **⊕** | **⊕** | **⊕** | **∅** | **⊕** | **⊕** | **∅** | **⊗** | **⊕** | **⊕** | **∅** | **⊗** | **⊗** | 5 |
| Steel *et al.* (2005) M.M. | **∅** | **⊕** | **⊕** | **⊕** | **∅** | **⊕** | **⊕** | **∅** | **⊗** | **⊕** | **⊕** | **∅** | **⊗** | **∅** | 5 |
| Bhui *et al.* (2006) C.D. | **⊕** | **⊕** | **⊕** | **⊗** | **∅** | **⊕** | **⊕** | **∅** | **⊗** | **⊕** | **⊕** | **∅** | **⊗** | **⊕** | 5 |
| Bhui *et al.* (2006) M.M. | **⊕** | **⊕** | **⊕** | **⊗** | **∅** | **⊕** | **⊕** | **∅** | **⊗** | **⊕** | **⊕** | **∅** | **⊗** | **⊕** | 5 |
| Roth & Ekblad (2006) C.D. | **⊕** | **∅** | **⊗** | **∅** | **∅** | **⊕** | **⊕** | **∅** | **⊗** | **⊗** | **⊕** | **∅** | **⊕** | **⊗** | 1 |
| Roth & Ekblad (2006) M.M. | **⊕** | **∅** | **⊗** | **∅** | **∅** | **⊕** | **⊕** | **∅** | **⊗** | **⊗** | **⊕** | **∅** | **∅** | **∅** | 1 |
| Schweitzer *et al.* (2006) C.D. | **⊕** | **⊕** | **∅** | **⊕** | **∅** | **⊕** | **⊕** | **∅** | **⊕** | **⊗** | **⊕** | **∅** | **⊗** | **⊕** | 6 |
| Schweitzer *et al.* (2006) M.M. | **⊕** | **⊕** | **∅** | **⊕** | **∅** | **⊕** | **⊕** | **∅** | **⊕** | **⊗** | **⊕** | **∅** | **⊗** | **⊕** | 6 |
| Ahmad *et al.* (2008) C.D. | **⊕** | **⊕** | **⊕** | **⊕** | **∅** | **⊕** | **⊕** | **∅** | **⊗** | **⊗** | **⊕** | **∅** | **⊗** | **⊗** | 3 |
| ***Table S3*** *continues on the next page* | | | | | | | | | | | | | | | |
| ***Table S3*** *continued* |  |  |  |  |  |  |  |  |  |  |  |  |  |  |  |
| Ahmad *et al.* (2008) M.M. | **⊕** | **⊕** | **⊕** | **⊕** | **∅** | **⊕** | **⊕** | **∅** | **⊗** | **⊗** | **⊕** | **∅** | **⊗** | **⊗** | 3 |
| Hodes *et al.* (2008) C.D. | **⊕** | **⊕** | **⊕** | **⊕** | **∅** | **⊕** | **⊕** | **∅** | **⊗** | **⊗** | **⊕** | **∅** | **⊗** | **⊗** | 3 |
| Hodes *et al.* (2008) M.M. | **⊕** | **∅** | **⊕** | **⊕** | **∅** | **⊕** | **⊕** | **∅** | **∅** | **⊗** | **⊕** | **∅** | **⊗** | **⊗** | 3 |
| Coffey *et al.* (2010) C.D. | **⊕** | **⊕** | **⊕** | **⊕** | **∅** | **⊕** | **⊕** | **∅** | **⊗** | **⊗** | **⊕** | **∅** | **⊗** | **⊕** | 5 |
| Coffey *et al.* (2010) M.M. | **⊕** | **⊕** | **⊕** | **⊕** | **∅** | **⊕** | **⊕** | **∅** | **⊗** | **⊗** | **⊕** | **∅** | **⊗** | **⊕** | 5 |
| Nickerson *et al.* (2010) C.D. | **⊕** | **⊕** | **⊕** | **⊕** | **∅** | **⊕** | **⊕** | **∅** | **⊗** | **⊗** | **⊕** | **∅** | **⊗** | **⊕** | 5 |
| Nickerson *et al.* (2010) M.M. | **⊕** | **⊕** | **⊕** | **⊕** | **∅** | **⊕** | **⊕** | **∅** | **⊗** | **⊗** | **⊕** | **∅** | **⊗** | **∅** | 4 |
| Silove *et al.* (2010) C.D. | **⊕** | **⊗** | **⊕** | **⊗** | **∅** | **⊕** | **⊕** | **∅** | **⊗** | **⊗** | **⊕** | **∅** | **⊗** | **⊗** | -1 |
| Silove *et al.* (2010) M.M. | **⊕** | **⊗** | **⊕** | **⊗** | **∅** | **⊕** | **⊕** | **∅** | **⊗** | **∅** | **⊕** | **∅** | **⊗** | **⊗** | 0 |
| Beiser *et al.* (2011) C.D. | **⊕** | **⊕** | **⊕** | **⊕** | **∅** | **⊕** | **⊕** | **∅** | **⊗** | **⊕** | **⊕** | **∅** | **⊗** | **⊕** | 7 |
| Beiser *et al.* (2011) M.M. | **⊕** | **⊕** | **∅** | **⊕** | **∅** | **⊕** | **⊕** | **∅** | **⊗** | **⊕** | **⊕** | **∅** | **⊗** | **∅** | 5 |
| Groark *et al.* (2011) C.D. | **⊕** | **⊕** | **⊕** | **⊕** | **∅** | **⊕** | **⊕** | **∅** | **⊗** | **⊕** | **⊕** | **∅** | **⊗** | **⊗** | 5 |
| Muhtz *et al.* (2011) C.D. | **⊕** | **⊕** | **⊕** | **⊕** | **∅** | **⊕** | **⊕** | **∅** | **∅** | **⊕** | **⊕** | **∅** | **⊗** | **⊗** | 6 |
| Muhtz *et al.* (2011) M.M. | **⊕** | **⊕** | **⊕** | **⊕** | **∅** | **⊕** | **⊕** | **∅** | **∅** | **⊕** | **⊕** | **∅** | **⊗** | **⊗** | 6 |
| Blair (2012) C.D. | **⊕** | **⊕** | **⊕** | **⊕** | **∅** | **⊕** | **⊕** | **∅** | **⊕** | **⊕** | **⊕** | **∅** | **⊗** | **⊕** | 9 |
| Blair (2012) M.M. | **∅** | **⊕** | **⊕** | **⊕** | **∅** | **⊕** | **⊕** | **∅** | **∅** | **⊕** | **⊕** | **∅** | **∅** | **⊕** | 8 |
| Bogic *et al.* (2012) C.D. | **⊕** | **⊕** | **⊕** | **⊗** | **∅** | **⊕** | **⊕** | **∅** | **⊕** | **⊕** | **⊕** | **∅** | **⊗** | **⊕** | 7 |
| Bogic *et al.* (2012) M.M. | **⊕** | **∅** | **⊕** | **⊗** | **∅** | **⊕** | **⊕** | **∅** | **⊕** | **∅** | **⊕** | **∅** | **⊗** | **⊕** | 5 |
| Heeren *et al.* (2012) C.D. | **⊕** | **⊕** | **⊕** | **⊗** | **∅** | **⊕** | **⊕** | **∅** | **⊕** | **⊕** | **⊕** | **∅** | **⊗** | **⊕** | 6 |
| Heeren *et al.* (2012) M.M. | **⊕** | **∅** | **⊕** | **⊗** | **∅** | **⊕** | **⊕** | **∅** | **⊕** | **⊕** | **⊕** | **∅** | **⊗** | **⊕** | 5 |
| Rasmussen *et al.* (2012) C.D. | **⊕** | **⊕** | **⊕** | **⊗** | **∅** | **⊕** | **⊕** | **∅** | **⊗** | **⊕** | **⊕** | **∅** | **⊗** | **⊕** | 5 |
| Rasmussen *et al.* (2012) M.M. | **⊕** | **⊕** | **⊕** | **∅** | **∅** | **⊕** | **⊕** | **∅** | **⊗** | **⊕** | **⊕** | **∅** | **∅** | **∅** | 6 |
| Warfa *et al.* (2012) C.D. | **⊕** | **⊗** | **⊕** | **⊗** | **∅** | **⊕** | **⊕** | **∅** | **⊗** | **⊕** | **⊕** | **∅** | **⊗** | **⊗** | 1 |
| Warfa *et al.* (2012) M.M. | **⊕** | **⊗** | **⊕** | **⊗** | **∅** | **⊕** | **⊕** | **∅** | **⊗** | **⊕** | **⊕** | **∅** | **⊗** | **⊗** | 1 |
| Bronstein *et al.* (2013) C.D. | **⊕** | **⊕** | **⊕** | **⊕** | **∅** | **⊕** | **⊕** | **∅** | **⊕** | **⊗** | **⊕** | **∅** | **⊗** | **⊗** | 5 |
| Bronstein *et al.* (2013) M.M. | **⊕** | **⊕** | **⊕** | **⊕** | **∅** | **⊕** | **⊕** | **∅** | **⊕** | **⊗** | **⊕** | **∅** | **⊗** | **⊗** | 5 |
| Cleveland & Rousseau (2013) C.D. | **⊕** | **⊕** | **⊕** | **⊗** | **∅** | **⊕** | **⊕** | **∅** | **⊗** | **⊗** | **⊕** | **∅** | **⊗** | **⊕** | 3 |
| Cleveland & Rousseau (2013) M.M. | **⊕** | **⊕** | **⊕** | **⊗** | **∅** | **⊕** | **⊕** | **∅** | **∅** | **⊗** | **⊕** | **∅** | **⊗** | **⊕** | 4 |
| Hollifield *et al.* (2013) C.D. | **⊕** | **⊕** | **⊕** | **⊗** | **∅** | **⊕** | **⊕** | **∅** | **⊕** | **⊗** | **⊕** | **∅** | **⊗** | **⊗** | 3 |
| Hollifield *et al.* (2013) M.M. | **⊕** | **⊕** | **⊕** | **⊗** | **∅** | **⊕** | **⊕** | **∅** | **⊕** | **⊗** | **⊕** | **∅** | **⊗** | **⊗** | 3 |
| Rees *et al.* (2013) C.D. | **⊕** | **⊗** | **⊕** | **⊗** | **∅** | **⊕** | **⊕** | **∅** | **⊗** | **⊗** | **⊕** | **∅** | **⊗** | **⊕** | 1 |
| Rees *et al.* (2013) M.M. | **∅** | **⊗** | **⊕** | **⊗** | **∅** | **⊕** | **⊕** | **∅** | **⊗** | **⊗** | **⊕** | **∅** | **∅** | **⊕** | 1 |
| Tay *et al.* (2013) C.D. | **⊕** | **⊕** | **⊕** | **⊗** | **∅** | **⊕** | **⊕** | **∅** | **⊕** | **⊕** | **⊕** | **∅** | **⊗** | **⊗** | 5 |
| Tay *et al.* (2013) M.M. | **⊕** | **⊕** | **⊕** | **⊗** | **∅** | **⊕** | **⊕** | **∅** | **⊕** | **⊕** | **⊕** | **∅** | **⊗** | **⊗** | 5 |
| ***Table S3*** *continues on the next page* | | | | | | | | | | | | | | | |
| ***Table S3*** *continued* |  |  |  |  |  |  |  |  |  |  |  |  |  |  |  |
| Heeren *et al.* (2014) C.D. | **⊕** | **⊕** | **⊕** | **⊗** | **∅** | **⊕** | **⊕** | **∅** | **⊕** | **⊗** | **⊕** | **∅** | **⊗** | **⊕** | 5 |
| Heeren *et al.* (2014) M.M. | **⊕** | **⊕** | **⊕** | **⊗** | **∅** | **⊕** | **⊕** | **∅** | **⊕** | **⊗** | **⊕** | **∅** | **⊗** | **⊕** | 5 |
| Lamkaddem *et al.* (2014) C.D. | **⊕** | **⊕** | **⊕** | **⊗** | **∅** | **⊕** | **⊕** | **∅** | **⊕** | **⊗** | **⊕** | **∅** | **⊕** | **⊕** | 7 |
| Lamkaddem *et al.* (2014) M.M. | **⊕** | **⊕** | **⊕** | **⊗** | **∅** | **⊕** | **⊕** | **∅** | **⊕** | **⊗** | **⊕** | **∅** | **⊕** | **⊕** | 7 |
| Mölsä *et al.* (2014) C.D. | **⊕** | **⊕** | **⊕** | **⊗** | **∅** | **⊕** | **⊕** | **∅** | **⊗** | **⊗** | **⊕** | **∅** | **⊗** | **⊕** | 3 |
| Mölsä *et al.* (2014) M.M. | **⊕** | **⊕** | **⊕** | **⊗** | **∅** | **⊕** | **⊕** | **∅** | **⊗** | **⊗** | **⊕** | **∅** | **⊗** | **∅** | 2 |
| Slewa-Younan *et al.* (2014) C.D. | **⊕** | **⊕** | **⊕** | **⊕** | **∅** | **⊕** | **⊕** | **∅** | **⊕** | **⊗** | **⊕** | **∅** | **⊗** | **⊗** | 5 |
| Slewa-Younan *et al.* (2014) M.M. | **⊕** | **⊕** | **⊕** | **⊕** | **∅** | **⊕** | **⊕** | **∅** | **⊕** | **⊗** | **⊕** | **∅** | **⊗** | **⊗** | 5 |
| Vervliet *et al.* (2014) C.D. | **⊕** | **⊕** | **⊕** | **⊗** | **∅** | **⊕** | **⊕** | **∅** | **⊗** | **⊗** | **⊕** | **∅** | **⊕** | **⊕** | 5 |
| Vervliet *et al.* (2014) M.M. | **⊕** | **⊕** | **⊕** | **⊗** | **∅** | **⊕** | **⊕** | **∅** | **⊗** | **⊗** | **⊕** | **∅** | **⊕** | **⊕** | 5 |
| Völkl-Kernstock *et al.* (2014) C.D. | **⊕** | **⊕** | **⊕** | **⊗** | **∅** | **⊕** | **⊕** | **∅** | **⊗** | **⊗** | **⊕** | **∅** | **⊗** | **⊗** | 1 |
| Völkl-Kernstock *et al.* (2014) M.M. | **⊕** | **⊕** | **⊕** | **⊗** | **∅** | **⊕** | **⊕** | **∅** | **∅** | **⊗** | **⊕** | **∅** | **⊗** | **⊗** | 0 |
| Hocking & Sundram (2015) C.D. | **⊕** | **⊕** | **⊕** | **⊗** | **∅** | **⊕** | **⊕** | **∅** | **⊕** | **⊗** | **⊕** | **∅** | **⊗** | **⊕** | 5 |
| Hocking & Sundram (2015) M.M. | **⊕** | **⊕** | **⊕** | **⊗** | **∅** | **⊕** | **⊕** | **∅** | **⊕** | **⊗** | **⊕** | **∅** | **⊗** | **⊕** | 5 |
| Jensen *et al.* (2015) C.D. | **⊕** | **⊕** | **⊕** | **⊗** | **∅** | **⊕** | **⊕** | **∅** | **⊗** | **⊗** | **⊕** | **∅** | **⊗** | **⊗** | 1 |
| Jensen *et al.* (2015) M.M. | **⊕** | **⊕** | **⊕** | **⊗** | **∅** | **⊕** | **⊕** | **∅** | **⊗** | **⊗** | **⊕** | **∅** | **⊗** | **⊗** | 1 |
| McGregor *et al.* (2015) C.D. | **⊕** | **⊗** | **∅** | **⊗** | **∅** | **⊕** | **⊕** | **∅** | **⊗** | **⊗** | **⊕** | **∅** | **⊗** | **⊗** | -2 |
| McGregor *et al.* (2015) M.M. | **⊕** | **⊗** | **∅** | **⊗** | **∅** | **⊕** | **⊕** | **∅** | **⊗** | **⊗** | **⊕** | **∅** | **∅** | **⊗** | -1 |
| Vonnahme *et al.* (2015) C.D. | **⊕** | **⊕** | **⊕** | **⊕** | **∅** | **⊕** | **⊕** | **∅** | **⊗** | **⊗** | **⊕** | **∅** | **⊗** | **⊕** | 5 |
| Vonnahme *et al.* (2015) M.M. | **⊕** | **⊕** | **⊕** | **⊕** | **∅** | **⊕** | **⊕** | **∅** | **⊗** | **⊗** | **⊕** | **∅** | **⊗** | **∅** | 4 |
| Morina *et al.* (2016) C.D. | **⊕** | **⊕** | **∅** | **⊕** | **∅** | **⊕** | **⊕** | **∅** | **⊕** | **⊗** | **⊕** | **∅** | **⊗** | **⊗** | 4 |
| Morina *et al.* (2016) M.M. | **⊕** | **⊕** | **∅** | **⊕** | **∅** | **⊕** | **⊕** | **∅** | **⊕** | **⊗** | **⊕** | **∅** | **⊗** | **⊗** | 4 |
| Park *et al.* (2017) C.D. | **⊕** | **⊕** | **⊕** | **⊗** | **∅** | **⊕** | **⊕** | **∅** | **⊗** | **⊗** | **⊕** | **∅** | **⊗** | **⊗** | 1 |
| Park *et al.* (2017) M.M. | **⊕** | **⊕** | **⊕** | **⊗** | **∅** | **⊕** | **⊕** | **∅** | **⊗** | **∅** | **⊕** | **∅** | **⊗** | **⊗** | 2 |
| Georgiadou *et al.* (2018) C.D. | **⊕** | **⊕** | **⊕** | **⊕** | **∅** | **⊕** | **⊕** | **∅** | **⊕** | **⊗** | **⊕** | **∅** | **⊗** | **⊕** | 7 |
| Georgiadou *et al.* (2018) M.M. | **⊕** | **⊕** | **⊕** | **⊕** | **∅** | **⊕** | **⊕** | **∅** | **⊕** | **⊗** | **⊕** | **∅** | **∅** | **⊕** | 6 |
| Javanbakht *et al.* (2018) C.D. | **⊕** | **⊕** | **⊕** | **⊕** | **∅** | **⊕** | **⊕** | **∅** | **⊕** | **⊗** | **⊕** | **∅** | **⊗** | **⊗** | 5 |
| Javanbakht *et al.* (2018) `M.M. | **⊕** | **∅** | **⊕** | **⊕** | **∅** | **⊕** | **⊕** | **∅** | **∅** | **⊗** | **⊕** | **∅** | **⊗** | **⊗** | 3 |
| Leiler *et al.* (2018) C.D. | **⊕** | **⊕** | **⊗** | **⊗** | **∅** | **⊕** | **⊕** | **∅** | **⊗** | **⊗** | **⊕** | **∅** | **⊗** | **⊗** | -1 |
| Leiler *et al.* (2018) M.M. | **⊕** | **∅** | **⊗** | **⊗** | **∅** | **⊕** | **⊕** | **∅** | **∅** | **⊗** | **⊕** | **∅** | **⊗** | **⊗** | -1 |
| Richter *et al.* (2018) C.D. | **⊕** | **⊕** | **⊕** | **⊕** | **∅** | **⊕** | **⊕** | **∅** | **⊗** | **⊕** | **⊕** | **∅** | **⊗** | **⊗** | 5 |
| Richter *et al.* (2018) M.M. | **⊕** | **⊕** | **∅** | **⊕** | **∅** | **⊕** | **⊕** | **∅** | **⊗** | **⊕** | **⊕** | **∅** | **⊗** | **⊗** | 4 |
| Schweitzer *et al.* (2018) C.D. | **⊕** | **⊕** | **⊕** | **⊗** | **∅** | **⊕** | **⊕** | **∅** | **⊕** | **⊗** | **⊕** | **∅** | **⊗** | **⊕** | 5 |
| Schweitzer *et al.* (2018) M.M. | **⊕** | **⊕** | **⊕** | **⊗** | **∅** | **⊕** | **⊕** | **∅** | **⊕** | **⊗** | **⊕** | **∅** | **⊗** | **⊕** | 5 |
| ***Table S3*** *continues on the next page* | | | | | | | | | | | | | | |  |
| ***Table S3*** *continued* |  |  |  |  |  |  |  |  |  |  |  |  |  |  |  |
| Kartal *et al.* (2019) C.D. | **⊕** | **⊕** | **⊕** | **⊕** | **∅** | **⊕** | **⊕** | **∅** | **⊕** | **⊗** | **⊕** | **∅** | **⊗** | **∅** | 6 |
| Kartal *et al.* (2019) M.M. | **⊕** | **∅** | **⊕** | **⊕** | **∅** | **⊕** | **⊕** | **∅** | **⊕** | **⊗** | **⊕** | **∅** | **⊗** | **⊕** | 6 |
| Poudel-Tandukar *et al.* (2019) C.D. | **⊕** | **⊕** | **⊕** | **⊕** | **∅** | **⊕** | **⊕** | **∅** | **⊕** | **⊗** | **⊕** | **∅** | **⊗** | **⊕** | 7 |
| Poudel-Tandukar *et al.* (2019) M.M. | **⊕** | **⊕** | **⊕** | **⊕** | **∅** | **⊕** | **⊕** | **∅** | **⊕** | **⊗** | **⊕** | **∅** | **⊗** | **⊕** | 7 |

C.D., assessed by Carla Deckers; M.M., assessed by Marc Molendijk

**⊕** = yes; **∅** = neutral / don’t know; **⊗** = no


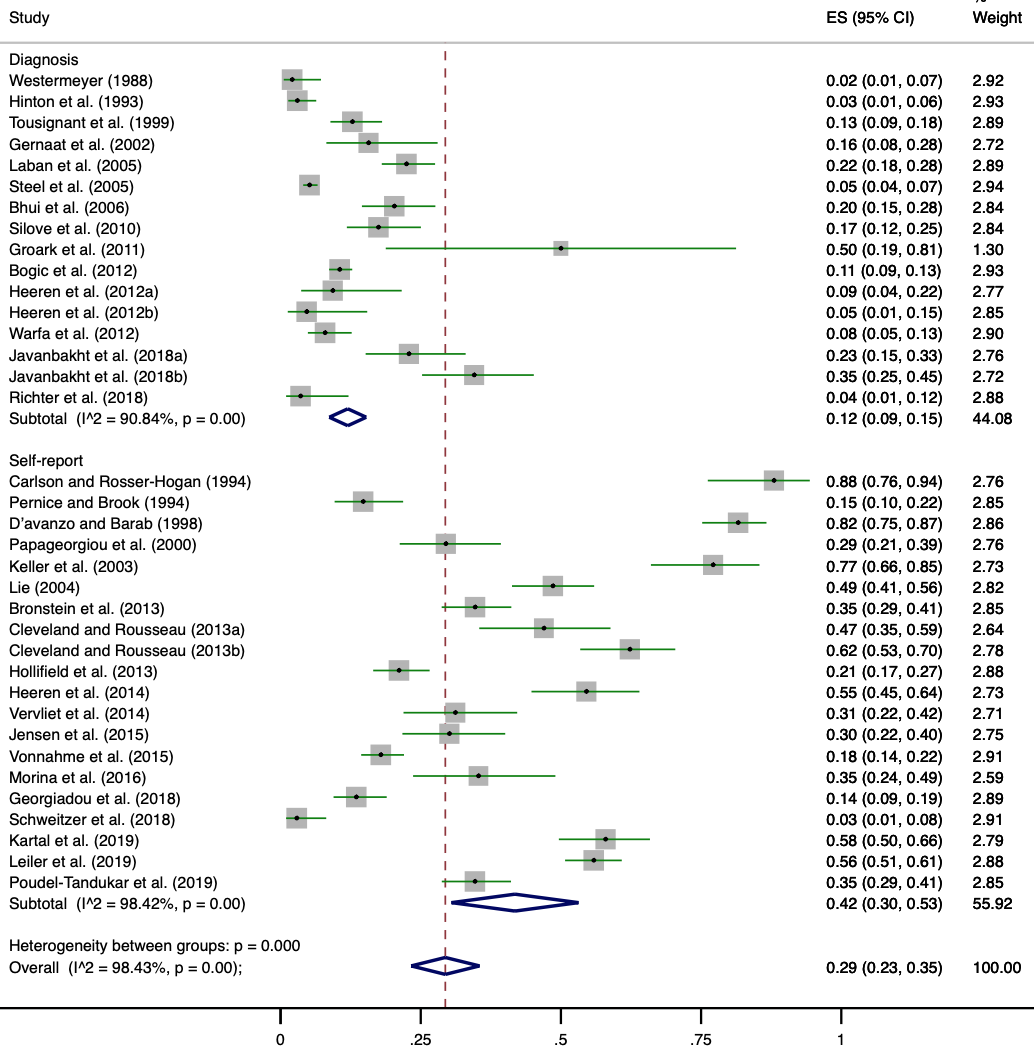
**Figure S1.** Forest plot of anxiety prevalence rates among refugees as assessed by diagnosis and self-report

**
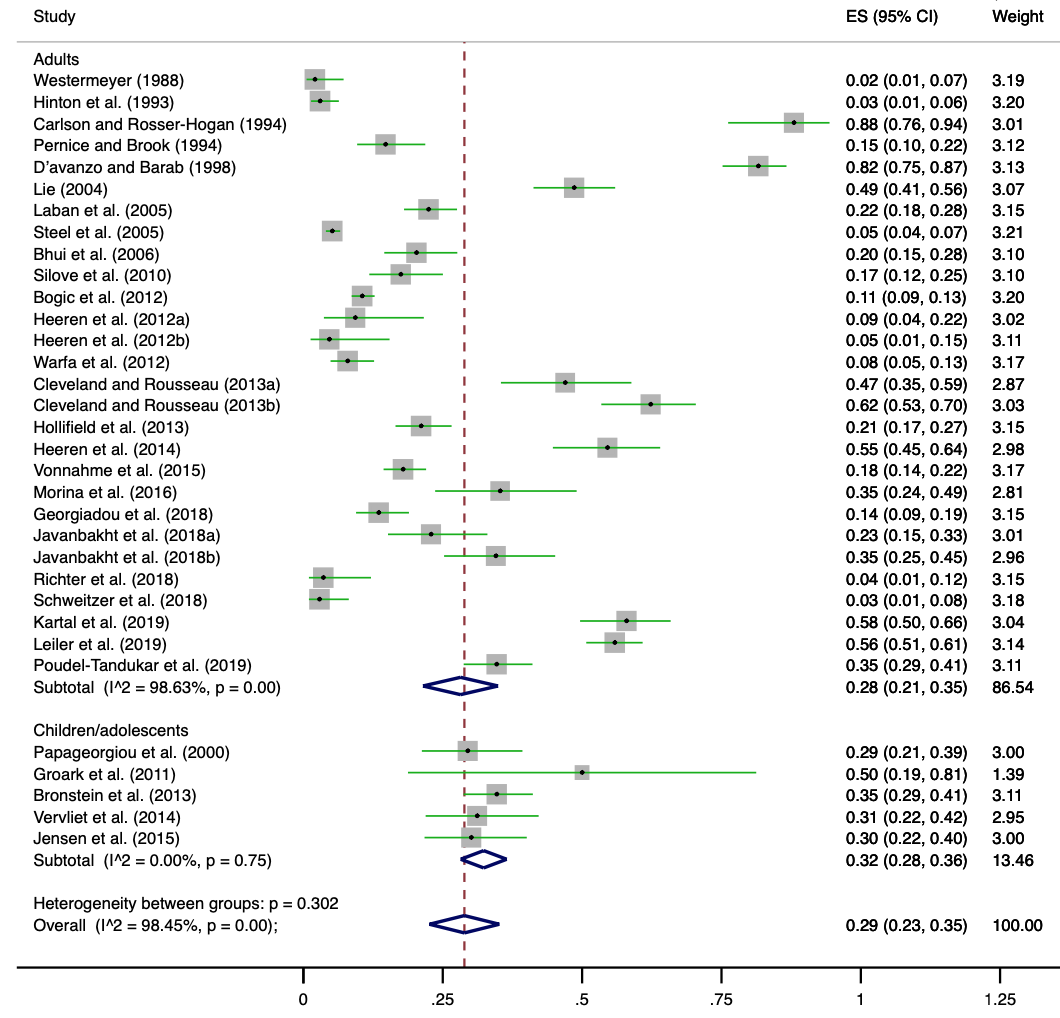
**

**Figure S2.** Forest plot of anxiety prevalence rates among adult and child/adolescent refugees


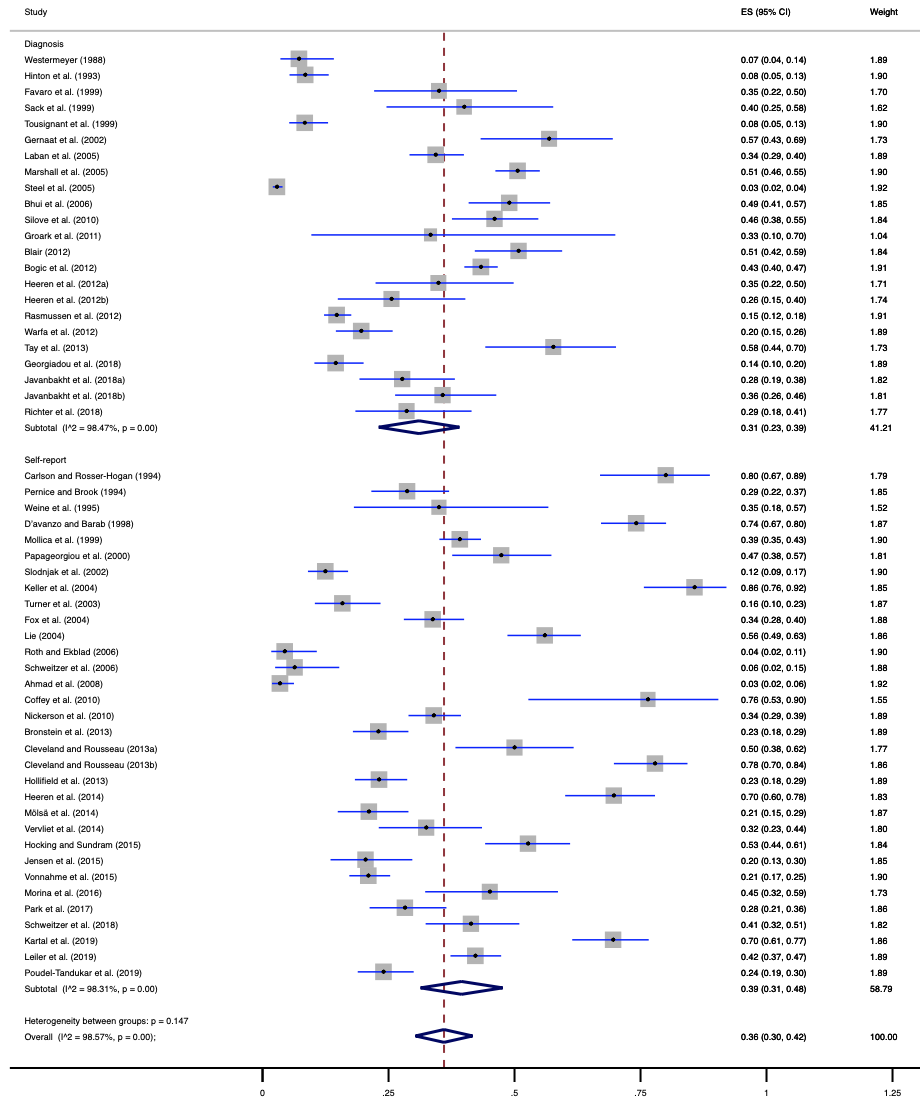
**Figure S3.** Forest plot of depression prevalence rates among refugees as assessed by diagnosis and self-report

**
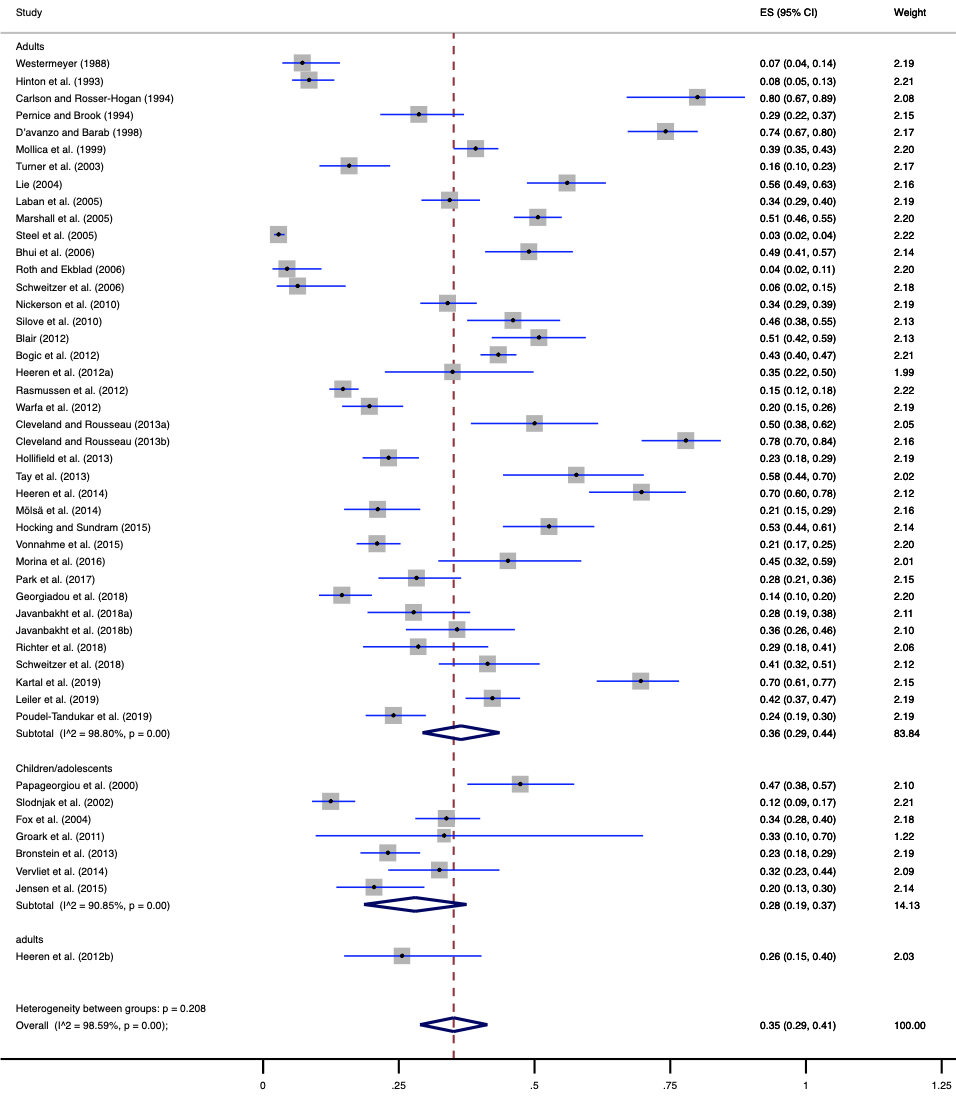
**

**Figure S4.** Forest plot of depression prevalence rates among adult and child/adolescent refugees

**
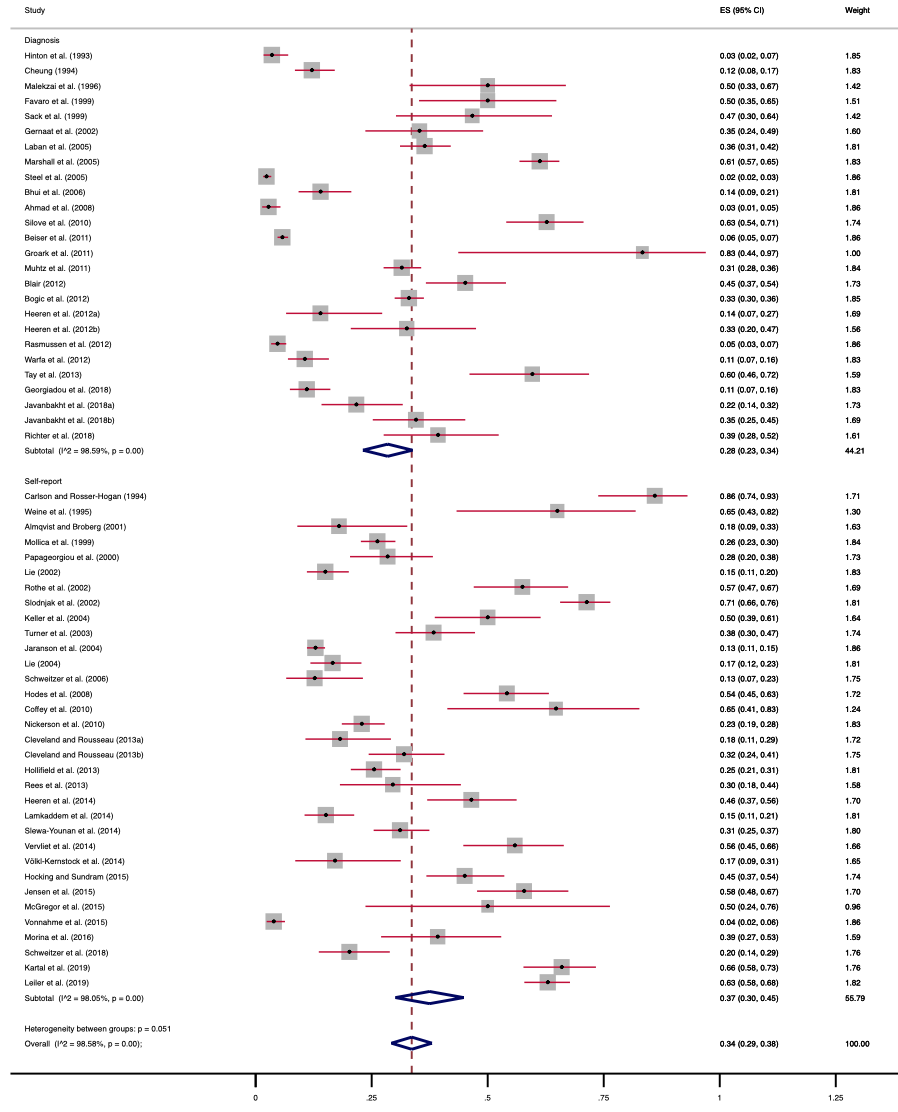
Figure S5.** Forest plot of PTSD prevalence rates as assessed by diagnosis and self-report


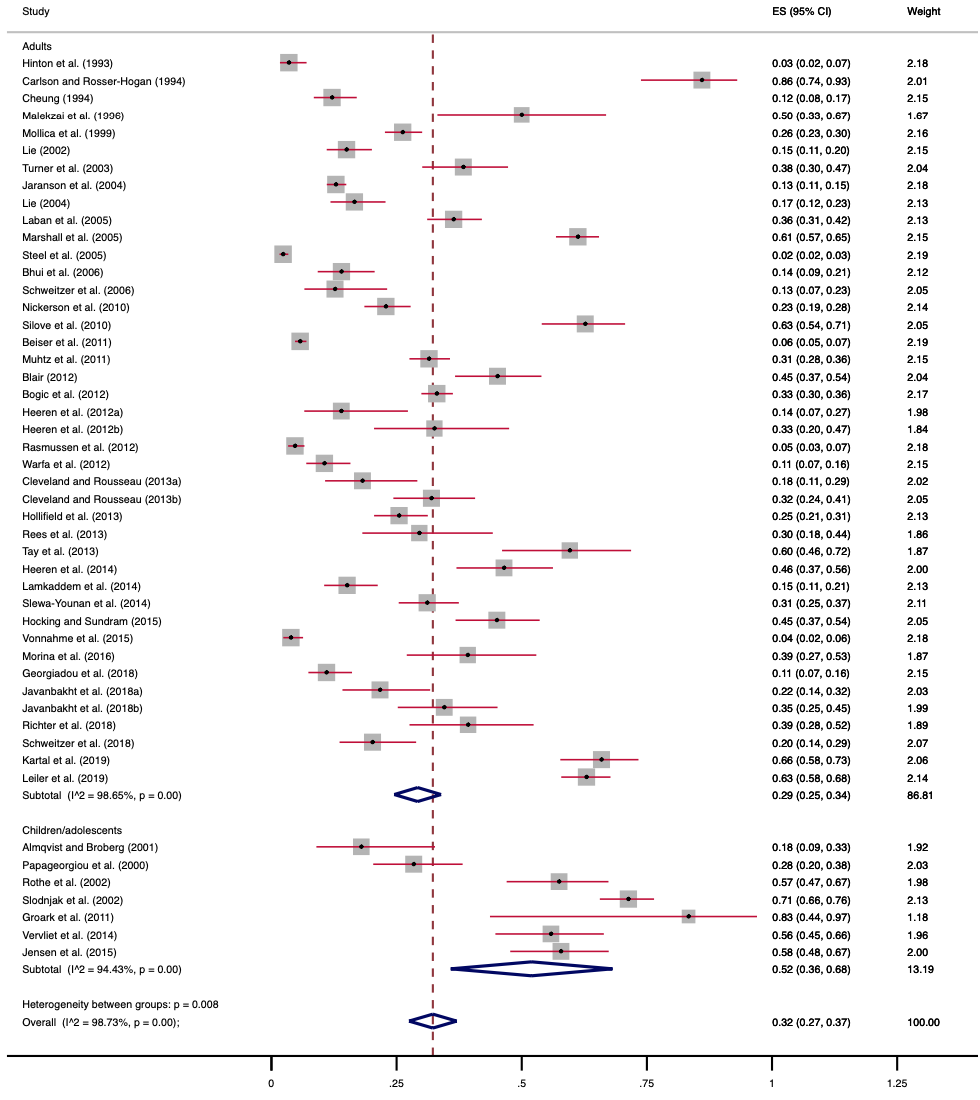


**Figure S6.** Forest plot of PTSD prevalence rates among adult and child/adolescent refugees

**Table S4.** Prevalence rates with 95% CI’s of anxiety, depression and PTSD by assessment method and adult *vs.* child/adolescent sample.

|  | Adult | *k* | *N* | *I^2^* | Child/adolescent | *k* | *N* | *I^2^* |
| --- | --- | --- | --- | --- | --- | --- | --- | --- |
| Anxiety | **0.28** (0.19-0.38) | 28 | 5,911 | 98.2*** | **0.32** (0.28-0.37) | 5 | 493 | 98.6*** |
| *Diagnosis* | **0.12** (0.07-0.17) ^a^ | 13 | 3,634 | 96.5*** | **0.50** | 1 | 6 | *N.A.* |
| *Self-report* | **0.42** (0.29-0.55) ^a^ | 15 | 2,277 | 98.6*** | **0.31** (0.28-0.36) | 4 | 487 | 0.0 |
| Depression | **0.36** (0.30-0.43) | 40 | 8,750 | 98.6*** | **0.28** (0.19-0.37) | 7 | 995 | 90.4*** |
| *Diagnosis* | **0.30** (0.22-0.38) | 18 | 4,900 | 98.3*** | **0.33** | 1 | 6 | *N.A.* |
| *Self-report* | **0.41** (0.31-0.50) | 22 | 3,850 | 98.3*** | **0.27** (0.18-0.38) | 6 | 989 | 92.3*** |
| PTSD | **0.29** (0.23-0.36) | 42 | 11,948 | 99.2*** | **0.52** (0.35-0.68) | 7 | 662 | 94.5*** |
| *Diagnosis* | **0.27** (0.19-0.36) | 21 | 7,161 | 99.4*** | **0.80** | 1 | 6 | *N.A.* |
| *Self-report* | **0.32** (0.23-0.40) | 21 | 4,787 | 98.5*** | **0.48** (0.32-0.65) | 6 | 656 | 94.5*** |

* *P* < .05, ** *P* < .01, *** *P* < .001

^a^ *Z*-value for difference in proportion = -1.76, *P* < .05

**Table S5.** Prevalence rates and 95% CI’s of anxiety, depression and PTSD for adult and child/adolescent refugees and (I) people living in conflict or war areas, (II) the general populations of Australia and New-Zealand, Europe and North America.

|  | Refugees | Conflict / war | Australia / N-Z | Europe | North America |
| --- | --- | --- | --- | --- | --- |
| Adult |  |  |  |  |  |
| Anxiety | **0.28** (0.19-0.38) | Not available | **0.06** (0.03-0.07) | **0.09** (0.05-0.13) | **0.18** (0.13-0.23) |
| Depression | **0.30** (0.22-0.38) | **0.09** (0.06-0.13) | **0.06** (0.04-0.08) | **0.04** (0.03-0.05) | **0.10** (0.07-0.13) |
| PTSD | **0.27** (0.19-0.36) | **0.11** (0.06-0.17) | **0.01** (0.01-0.02) | **0.01** (0.01-0.02) | **0.04** (0.02-0.06) |
| Child/adolescent |  |  |  |  |  |
| Anxiety | **0.32** (0.28-0.37) | **0.27** (0.21-0.33) | **0.03** (0.04-0.09) | **0.03** (0.01-0.05) | **0.03** (0.01-0.06) |
| Depression | **0.28** (0.19-0.37) | **0.43** (0.31-0.55) | **0.07** (0.02-0.04) | **0.02** (0.02-0.04) | **0.03** (0.02-0.06) |
| PTSD | **0.52** (0.36-0.68) | **0.47** (0.35-0.60) | Not available | Not available | **0.05** (0.01-0.10) |

*Note*. Where possible we chose to report 1-year prevalence estimates.

Prevalence estimates on refugees are derived from this study. For the purpose of comparability among samples, we choose estimates derived through a diagnostic interview for adult samples. Due to a lack of sufficient data, we used estimates derived from diagnostic interview, and parent and self-report for child and adolescent estimates. The estimates on prevalence rates in conflict and war settings are derived from Charlson *et al.,* 2019 (adults) and Attanayake *et al.,* 2009 (children and adolescents). Adult prevalence estimates on depression and anxiety for Australia / New-Zealand, Europe, and North America come from Baumeister and Härter, 2007. The estimates on PTSD prevalence in Australia / New-Zealand also come from this source. Adult PTSD prevalence estimates in Europe and North America come from Alonso *et al.,* 2004. The estimates on childhood depression and anxiety prevalence in Australia come from Ciobanu *et al.,* 2018. Childhood depression-, anxiety-, and PTSD prevalence are derived from Merikangas, Nakamura, and Kessler, 2009.

For adults, refugee prevalence estimates differ significantly (*P* < .05) from estimates in all other cells per row. Childhood prevalence estimates differ from those reported in Australia, Europe and North America (*P* < .05), but not from those reported in conflict and war areas.

**Table S6.** Prevalence rates with 95% CI’s of anxiety, depression and PTSD by assessment method and by continent of origin.

|  | Africa [*k*] | Asia [*k*] | Europe [*k*] | Other/mix [*k*] |
| --- | --- | --- | --- | --- |
| Anxiety | **0.19** (0.06-0.32)[3] | **0.29** (0.17-0.40)[17] | **0.34** (0.07-0.62)[3] | **0.33** (0.18-0.47)[13] |
| *Diagnosis* | --- ^a^ | **0.15** (0.07-0.23)[8] | --- ^a^ | **0.08** (0.03-0.13)[5] |
| *Self-report* | --- ^a^ | **0.40** (0.22-0.58)[9] | --- ^a^ | **0.43** (0.27-0.61)[8] |
| Depression | **0.26** (0.14-0.38)[6] | **0.35** (0.26-0.43)[23] | **0.34** (0.15-0.53)[6] | **0.41** (0.31-0.52)[20] |
| *Diagnosis* | --- ^a^ | **0.31** (0.20-0.42)[12] | --- ^a^ | **0.29** (0.17-0.40)[8] |
| *Self-report* | **0.22** (0.08-0.34)[4] | **0.39** (0.26-0.53)[11] | **0.32** (0.10-0.55)[5] | **0.49** (0.35-0.63)[12] |
| PTSD | **0.24** (0.02-0.46)[4] | **0.33** (0.26-0.42)[24] | **0.41** (0.29-0.54)[5] | **0.36** (0.28-0.43)[26] |
| *Diagnosis* | --- ^a^ | **0.30** (0.19-0.41)[14] | --- ^a^ | **0.34** (0.15-0.53)[8] |
| *Self-report* | --- ^a^ | **0.37** (0.20-0.53)[10] | **0.48** (0.30-0.66)[3] | **0.36** (0.28-0.45)[18] |

* *P* < .05, ** *P* < .01, *** *P* < .001

^a^ Not enough data (i.e., ≥ 2 effect-sizes) to calculate pooled estimates.

*Note.* *Z*-value for differences in proportions = -1.96, *P* < .05

**Table S7.** Prevalence rates with 95% CI’s of anxiety, depression and PTSD by assessment method and by continent of resettlement.

|  | Australia/NZ [*k*] | Europe [*k*] | U.S.A./Can [*k*] | Other/mix [*k*] |
| --- | --- | --- | --- | --- |
| Anxiety | **0.19** (0.01-0.39)[5] | **0.28** (0.19-0.36)[16] | **0.35** (0.19-0.51)[12] | **0.33** (-0.14-0.81)[3] |
| *Diagnosis* | --- ^a^ | **0.13** (0.07-0.21)[7] | **0.14** (0.03-0.26)[5] | --- ^a^ |
| *Self-report* | **0.25** (-0.10-0.58)[3] | **0.37** (0.28-0.46)[9] | **0.50** (0.29-0.70)[7] | --- ^a^ |
| Depression | **0.41** (0.26-0.56)[10] | **0.32** (0.25-0.39)[23] | **0.37** (0.26-0.49)[18] | **0.41** (0.18-0.64)[4] |
| *Diagnosis* | **0.35** (0.02-0.68)[3] | **0.32** (0.22-0.41)[10] | **0.27** (0.15-0.39)[9] | --- ^a^ |
| *Self-report* | **0.43** (0.26-0.61)[7] | **0.31** (0.20-0.41)[13] | **0.48** (0.30-0.65)[9] | --- ^a^ |
| PTSD | **0.33** (0.22-0.44)[14] | **0.34** (0.26-0.42)[24] | **0.34** (0.23-0.45)[18] | **0.36** (0.05-0.68)[3] |
| *Diagnosis* | **0.34** (0.03-0.65)[4] | **0.29** (0.18-0.41)[11] | **0.30** (0.15-0.44)[8] | --- ^a^ |
| *Self-report* | **0.33** (0.22-0.43)[10] | **0.38** (0.27-0.49)[13] | **0.38** (0.22-0.54)[10] | --- ^a^ |

^a^ Not enough data (*i.e.,* ≥ 2 effect-sizes) to calculate pooled estimates.

**Table S8.** Time related trends in prevalence rates of anxiety, depression and PTSD by assessment method.

|  | Year - prevalence | Year – prevalence [diagnosis] | Year – prevalence [self-report] |
| --- | --- | --- | --- |
| All | *r* = -.01 (*k* = 150) | *r* = .16 (*k* = 65) | *r* = -.16 (*k* = 85) |
| Anxiety | *r* = .03 (*k* = 36) | *r* = .38 (*k* = 16) | *r* = -.34 (*k* = 20) |
| Depression | *r* = .05 (*k* = 55) | *r* = .38 (*k* = 16) | *r* = -.08 (*k* = 32) |
| PTSD | *r* = -.07 (*k* = 59) | *r* = -.01 (*k* = 26) | *r* = -.14 (*k* = 33) |

^b^ *Z*-value for difference in proportion = -1.96, *P* < .05

**Table S9.** Results of moderation analyses by outcome and assessment method.

|  | Age | % female | Duration of stay | Methodological quality |
| --- | --- | --- | --- | --- |
| Anxiety | >-0.0004 < 0 | 0.0002 | 0.0005 | -0.02 |
| *Diagnosis* | 0.006 | 0.001 | >-0.0005 < 0 | -0.005 |
| *Self-report* | 0.004 | <0.0009 > 0 | 0.001 | -0.01 |
| Depression | 0.01 | 0.001 | >-0.0001 < 0 | 0.01 |
| *Diagnosis* | 0.006 | 0.002 | >-0.0008 < 0 | 0.003 |
| *Self-report* | 0.007 | <0.0001 > 0 | <0.0005 > 0 | 0.03 |
| PTSD | <0.0001 > 0 | >-0.0001 < 0 | >-0.0001 and < 0 | -0.02 |
| *Diagnosis* | <0.001 > 0 | 0.001 | >-0.0001 and < 0 | -0.004 |
| *Self-report* | -0.004 | -0.001 | 0.003 | -0.03 |

* *P* < .05, ** *P* < .01, *** *P* < .001

**Table S10.** Associations among moderator variables.

|  | Age | % female | Dur. stay | Instrument | Met. quality | Origin | Resettlement |
| --- | --- | --- | --- | --- | --- | --- | --- |
| Age |  | 0.26* | 0.35** | 4.50 | 0.21 | 4.15* ^A^ | 0.08 |
| % female | 0.26* |  | 0.12 | 0.08 | -0.03 | 0.11 | 2.63 |
| Dur. of stay | 0.35** | 0.12 |  | 1.82 | 0.09 | 4.63* ^B^ | 0.04 |
| Instrument | 4.50 | 0.08 | 1.82 |  | 3.12 | 0.79 | 0.31 |
| Met. Quality | 0.21* | -0.03 | 0.09 | 3.12 |  | 1.79 | 1.03 |
| Origin | 4.15* ^A^ | 0.11 | 4.63* ^B^ | 0.79 | 1.79 |  | 0.13 |
| Resettlement | 0.08 | 2.63 | 0.04 | 0.31 | 1.03 | 0.13 |  |

* *P* < .05, ** *P* < .01

*Note.* Associations among continuous variables were assessed by means of correlation coefficients. Associations among categorical variables were assessed by means of chi-square tests and Lambda statistics. Associations among categorical- and continuous variables were assessed by means of analyses of variance *F*-tests.

^A^ Refugees from Europe on average were older relative to refugees from other continents

^B^ Refugees from Europe on average were in the country of resettlement for a longer period of time

**References**

Westermeyer J. DSM-III psychiatric disorders among Hmong refugees. *Am J Psychiatry* 1988; **145(2):** 197-202.

Hinton WL, Chen YCJ, Du N, et al. DSM-III—R disorders in Vietnamese refugees: Prevalence and correlates. J Nerv Ment Dis 1993; **181(2):** 113-22.

Carlson EB, Rosser-Hogan R. Cross-cultural response to trauma: A study of traumatic experiences and posttraumatic symptoms in Cambodian refugees. J Traumatic Stress 1994; **7(1):** 43-58.

Cheung, P. Posttraumatic stress disorder among cambodian refugees in New Zealand. Int J Soc Psychiatry 1994; **40(1):** 17-26.

Pernice R, Brook J. Relationship of migrant status (refugee or immigrant) to mental health. Int J Soc Psychiatry 1994; **40(3):** 177-88.

Weine SM, Becker DF, McGlashan TH, et al. Psychiatric consequences of 'ethnic cleansing': Clinical assessments and trauma testimonies of newly resettled Bosnian refugees. Am J Psychiatry 1995; **152(4):** 536-42.

Malekzai ASB, Niazi JM, Paige SR, et al. Modification of CAPS-1 for diagnosis of PTSD in Afghan refugees. J Trauma Stress 1996; **9(4):** 891-8.

D'Avanzo CE, Barab SA. Depression and anxiety among Cambodian refugee women in France and the United States. Issues Ment Health Nurs 1998; **19(6):** 541-56.

Almqvist K, Broberg AG. Mental health and social adjustment in young refugee children y 3½ years after their arrival in Sweden. J Am Acad Child Adolesc Psychiatry 1999; **38(6):** 723-30.

Mollica R, Caspi-Yavin Y, Bollini P, et al. The Harvard Trauma Questionnaire. J Nerv Ment Dis 1992;

**180(2):** 111-6.

Sack WH, Him C, Dickason D. Twelve‐year follow‐up study of khmer youths who suffered massive war trauma as children. J Am Acad Child Adolesc Psychiatry 1999; **38(9):** 1173-9.

Tousignant M, Habimana E, Biron C, et al. The Quebec adolescent refugee project: psychopathology and family variables in a sample from 35 nations. J Am Acad Child Adolesc Psychiatry 1999; **38(11):** 1426-1432.

Papageorgiou V, Frangou-Garunovic A, Iordanidou R, et al. War trauma and psychopathology in Bosnian refugee children. Eur Child Adolesc Psychiatry 2000; **9(2):** 84-90.

Bayard-Burfield L, Sundquist J, Johansson SE. Ethnicity, self-reported psychiatric illness, and intake of psychotropic drugs in five ethnic groups in Sweden. *J Epidem Comm Health* 2001; **55(9):** 657-664.

Gernaat H, Malwand A, Laban C, et al. Veel psychiatrische stoornissen bij Afghaanse vluchtelingen met verblijfsstatus in Drenthe, met name depressieve stoornis en posttraumatische stressstoornis. Ned Tijdschr Geneeskd 2002; **146(24):** 1127-31.

Lie B. A 3‐year follow‐up study of psychosocial functioning and general symptoms in settled refugees. Acta Psychiatr Scand 2002; **106(6):** 15-425.

Rothe EM, Lewis J, Castillo-Matos H, et al. Posttraumatic stress disorder among Cuban children and adolescents after release from a refugee camp. Psychiatr Serv 2002; **53(8):** 970-6.

Slodnjak V, Kos A, Yule W. Depression and parasuicide in refugee and Slovenian adolescents. Crisis: J Crisis Interven Suicide Prevention 2002; **23(3):** 127.

Keller AS, Rosenfeld B, Trinh-Shevrin C, et al. Mental health of detained asylum seekers. Lancet 2003; **362(9397):** 1721-3.

Turner SW, Bowie C, Dunn G, et al. Mental health of kosovan albanian refugees in the UK. The Br J Psychiatry 2003; **182(5):** 444-8.

Fox PG, Burns KR, Popovich JM, et al. Southeast Asian refugee children: Self-esteem as a predictor of depression and scholastic achievement in the US. Int J Psychiatric Nursing Res 2004; **9(2):** 1063-72

Jaranson JM, Butcher J, Halcon L, et al. Somali and Oromo refugees: Correlates of torture and trauma history. Am J Pub Health 2004; **94(4):** 591-8.

Lie B. The psychological and social situation of repatriated and exiled refugees: A longitudinal, comparative study. Scand J Public Health 2004; **32(3):** 179-187.

Laban CJ, Gernaat HB, Komproe IH, et al. Post-migration living problems and common psychiatric disorders in Iraqi asylum seekers in the Netherlands. J Nerv Ment Dis 2005; **193(12):** 825-32.

Marshall GN, Schell TL, Elliott MN, et al. Mental health of cambodian refugees 2 decades after resettlement in the united states. J Am Med Assoc 2005; **294(5):** 571-9.

Bhui K, Craig T, Mohamud S, et al. Mental disorders among Somali refugees: Developing culturally appropriate measures and assessing socio-cultural risk factors. Soc Psychiatry Psychiatric Epidem 2006; **41(5):** 400-8.

Roth G, Ekblad S. A longitudinal perspective on depression and sense of coherence in a sample of mass-evacuated adults from Kosovo. J Nerv Ment Dis 2006; **194(5):** 378-81.

Schweitzer R, Melville F, Steel Z. et al. Trauma, post-migration living difficulties, and social support as predictors of psychological adjustment in resettled Sudanese refugees. Aust N Z J Psychiatry 2006; **40(2):** 179-87.

Ahmad A, von Knorring A, Sundelin-Wahlsten V. Traumatic experiences and post-traumatic stress disorder in Kurdistanian children and their parents in homeland and exile: An epidemiological approach. Nord J Psychiatry 2008; **62(6):** 457-63.

Hodes M, Jagdev D, Chandra N, et al. Risk and resilience for psychological distress amongst unaccompanied asylum seeking adolescents. J Child Psychol Psychiatry 2008; **49(7):** 723-32.

Coffey GJ, Kaplan I, Sampson RC, et al. The meaning and mental health consequences of long-term immigration detention for people seeking asylum. Soc Sci Med 2010; **70(12):** 2070-9.

Nickerson A, Bryant RA, Steel Z, et al. The impact of fear for family on mental health in a resettled Iraqi refugee community. J Psychiatric Res 2010; **44(4):** 229-35.

Silove D, Momartin S, Marnane C, et al. Adult separation anxiety disorder among war‐affected Bosnian refugees: Comorbidity with PTSD and associations with dimensions of trauma. J Traumatic Stress 2010; **23(1):** 169-72.

Beiser M, Simich L, Pandalangat N, et al. Stresses of passage, balms of resettlement, and posttraumatic stress disorder among Srilankan Tamils in Canada. Can Psychiatry 2011; **56(6):** 333-40.

Groark C, Sclare I, Raval H. Understanding the experiences and emotional needs of unaccompanied asylum-seeking adolescents in the UK. Clin Child Psychol Psychiatry 2011; **16(3):** 421-42.

Muhtz C, von Alm C, Godemann K, et al. Langzeitfolgen von in der Kindheit am ende des II. Weltkrieges erlebter Flucht und Vertreibung. Psychotherapie Psychosomatik· Medizinische Psychologie 2011; **61(05):** 233-38.

Blair RG. Risk factors associated with PTSD and major depression among Cambodian refugees in Utah. Health & Social Work 2000; **25(1):** 23-30.

Bogic M, Ajdukovic D, Bremner S, et al. Factors associated with mental disorders in long-settled war refugees: Refugees from the former Yugoslavia in Germany, Italy and the UK. Br J Psychiatry 2102; **200(3):** 216-23.

Heeren M, Mueller J, Ehlert U, et al. Mental health of asylum seekers: A cross-sectional study of psychiatric disorders. BMC Psychiatr 2012; **12(1):** 114.

Rasmussen A, Crager M, Baser RE, et al. Onset of posttraumatic stress disorder and major depression among refugees and voluntary migrants to the united states. J Traumatic Stress 2012; **25(6):** 705-12.

Warfa N, Curtis S, Watters C, et al. Migration experiences, employment status and psychological distress among Somali immigrants: A mixed-method international study. BMC Public Health 2012; **12(1):** 749.

Bronstein I, Montgomery P, Ott, E. Emotional and behavioural problems amongst afghan unaccompanied asylum-seeking children: Results from a large-scale cross-sectional study. Eur Child Adoles Psychiatry 2013; **22(5):** 285-94.

Cleveland J, Rousseau C. Psychiatric symptoms associated with brief detention of adult asylum seekers in Canada. Can J Psychiatry 2013; **58(7):** 409-16.

Hollifield M, Verbillis-Kolp S, Farmer B, et al. The refugee health screener-15: development and validation of an instrument for anxiety, depression, and PTSD in refugees. Gen Hospital Psychiatry 2013; **35(2):** 202-9.

Rees S, Silove DM, Tay K, et al. Human rights trauma and the mental health of west Papuan refugees resettled in Australia. Med J Australia 2013; **199(4):** 280-3.

Tay K, Frommer N, Hunter J, et al. A mixed-method study of expert psychological evidence submitted for a cohort of asylum seekers undergoing refugee status determination in Australia. Soc Sci Med 2013; **98:** 106-15.

Heeren M, Wittmann L, Ehlert U, et al. Psychopathology and resident status–comparing asylum

seekers, refugees, illegal migrants, labor migrants, and residents. Comprehen Psychiatry

2014; **55(4):** 818-25.

Lamkaddem M, Stronks K, Devillé WD, et al. Course of post-traumatic stress disorder and health care utilisation among resettled refugees in the Netherlands. BMC Psychiatry 2014; **14(1):** 90.

Mölsä M, Punamäki R, Saarni SI, et al. (2014). Mental and somatic health and pre-and post-migration factors among older Somali refugees in Finland. Transcult Psychiatry 2014; **51(4):** 499-525

Vervliet M, Lammertyn J, Broekaert E, et al. Longitudinal follow-up of the mental health of unaccompanied refugee minors. Eur Child Adolescent Psychiatr 2014; **23(5):** 337-46.

Völkl-Kernstock S, Karnik N, Mitterer-Asadi M, et al. Responses to conflict, family loss and flight: Posttraumatic stress disorder among unaccompanied refugee minors from Africa. Neuropsychiatry 2014; **28(1):** 6-11.

Hocking D, Sundram S. Demoralisation syndrome does not explain the psychological profile of community-based asylum-seekers. Comprehen Psychiatry 2015; **63:** 55-64.

Jensen TK, Fjermestad KW, Granly L, et al. Stressful life experiences and mental health problems among unaccompanied asylum-seeking children. Clin Child Psychol Psychiatry 2015; **20(1):** 106-16.

McGregor LS, Melvin GA, Newman LK. Differential accounts of refugee and resettlement experiences in youth with high and low levels of posttraumatic stress disorder symptomatology: a mixed-methods investigation. Am J Orthopsychiatry 2015; **85(4):** 371.

Vonnahme LA, Lankau EW, Ao T, et al. Factors associated with symptoms of depression among Bhutanese refugees in the united states. J Imm Minority Health 2015; **17(6):** 1705-14.

Morina N, Sulaj V, Schnyder U, et al. Obsessive-compulsive and posttraumatic stress symptoms among civilian survivors of war. BMC Psychiatry 2016; **16(1):** 115.

Park S, Lee M, Jeon J. Factors affecting depressive symptoms among North Korean adolescent refugees residing in South Korea. Int J Environ Res Public Health 2017; **14(8):** 912.

Georgiadou E, Zbidat A, Schmitt GM, et al. (2018). Prevalence of mental distress among Syrian refugees with residence permission in Germany: A registry-based study. Front Psychiatry 2018; **9:** 393.

Javanbakht A, Rosenberg D, Haddad L, et al. Mental health in Syrian refugee children resettling in the united states: War trauma, migration, and the role of parental stress. Am J of Child and Adolesc Psychiatry 2018; **57(3):** 209-11.

Richter K, Peter L, Lehfeld H, et al. Prevalence of psychiatric diagnoses in asylum seekers with follow-up. BMC Psychiatry 2018; **18(1):** 206.

Schweitzer RD, Vromans L, Brough M, et al. Recently resettled refugee women-at-risk in australia evidence high levels of psychiatric symptoms: Individual, trauma and post-migration factors predict outcomes. BMC Med 2018; **16(1):** 149.

Kartal D, Alkemade N, Kiropoulos L. Trauma and mental health in resettled refugees: mediating effect of host language acquisition on posttraumatic stress disorder, depressive and anxiety symptoms. Transcult Psychiatry 2019; **56(1):** 3-23.

Leiler A, Bjärtå A, Ekdahl J,et al. Mental health and quality of life among asylum seekers and refugees living in refugee housing facilities in Sweden. Soc Psychiatry Psychiatric Epidem 2019; **54(5):** 543-51.

Poudel-Tandukar K, Chandler GE, Jacelon CS, et al. Resilience and anxiety or depression among resettled Bhutanese adults in the United States. Int J Soc Psychiatry 2019; **65(6):** 496-506.
